# Supplementary material for: Profiling cell envelope-antibiotic interactions reveals vulnerabilities to β-lactams in a multidrug-resistant bacterium
Source: Nat Commun. 2023 Aug 9;14:4815. doi: 10.1038/s41467-023-40494-5 (PMC10412643; doi:10.1038/s41467-023-40494-5)
Supplement: Supplementary file 1 — Supplementary Information [file 41467_2023_40494_MOESM1_ESM.pdf]

Supplementary information for:

Hogan et al., Profiling cell envelope-antibiotic interactions reveals vulnerabilities to β-lactams in a multidrug-resistant bacterium

Supplementary Tables S1 – S12 are given here

Supplementary Figures S1 – S13 are given here

Supplementary Data 1 and 2 are provided as separate Excel files

Supplementary Table 1 Summary statistics of the randomly barcoded transposon mutant library

Category	K56-2 Library
Number of mutant colonies recovered	~815, 000
Number of unique barcodes	338,116
Number of unique insertion positions	145,585
Protein-coding genes with (or without) central insertions (10-90% of gene)	6412 (561)
Mean/median strains per interrupted protein-coding gene	31.9 / 12
Gene and transposon on same strand	49.9%
Average distance between insertions	18.7 bp

Supplementary Table 2. Fold change in targeted gene expression of associated CRISPRi mutants induced with rhamnose used in this work (listed in order of appearance).

Target Gene/Operon	Fold Change vs. NTC (± SD)	Associated Figure
mlaFEDvacJ	-3.6 ± 0.1	1
mlaCB	-17.8 ± 0.8	1
hldD	-25.9 ± 1.9	2
wabR-P	-79.4 ± 5.8	2
wbiFGHI	-5.8 ± 0.4	2
vioAwbxFED	-20.2 ± 1.0	2
ogcABEI	-9.7 ± 0.3	2
pglL	-6.4 ± 0.2	2
ispDF	-3.0 ± 0.1	2, 3, and 4
uppSrseP	-3.5 ± 0.2	2, 3, and 4
bla_{PenR}	-1.1 ± 0.1	5
bla_{PenB}	-1.6 ± 0.1	5
bla_{AmpC}	-35.6 ± 1.9	5
K562_RS32470	-3.8 ± 0.2	5

NTC = non-targeting control sgRNA

Supplementary Table 3. Effect of CRISPRi knockdown of β-lactamase genes on susceptibility to AMP, CFD, TAZ, and CTT. MIC values are the median of three replicates and are reported in µg/mL. Bolded values indicate at least 2-fold change.

		MIC (µg/mL)		Fold
Antibiotic	Gene	-Rha	+Rha	MIC*
AMP	NTC	>512	>512	-
	penR	>512	>512	-
	bla$_{\mathrm{AmpC}}$	>512	>512	-
	bla$_{\mathrm{PenB}}$	>512	**256**	**>2**
CFD	NTC	0.125	0.125	1
	penR	0.125	0.125	1
	bla$_{\mathrm{AmpC}}$	0.125	0.125	1
	bla$_{\mathrm{PenB}}$	0.125	0.125	1
TAZ	NTC	128	128	1
	penR	128	**64**	**2**
	bla$_{\mathrm{AmpC}}$	128	128	**1**
	bla$_{\mathrm{PenB}}$	128	**32**	**4**
CTT	NTC	64	64	1
	penR	64	**16**	**4**
	bla$_{\mathrm{AmpC}}$	64	64	1
	bla$_{\mathrm{PenB}}$	64	64	1

*Fold MIC is the ratio of the MIC -Rha to the MIC +Rha

Supplementary Table 4. MIC values of other β-lactams in K56-2 with AVI and TAZ. MIC values are the median of three replicates and are reported in µg/mL. Bolded values indicate changes upon β-lactamase addition.

	**β-Lactam Alone**	**+ 8 µg/mL AVI**	**+ 8 µg/mL TAZ**
Ceftazidime	32	**4**	32
Cefotaxime	>128	**16**	>128
Cephalexin	>128	**128**	>128
Cefmetazole	128	128	128
Cefoperazone	>128	**64**	>128
Ceftriaxone	>128	**4**	>128
Moxalactam	128	128	128

Supplementary Table 5. Susceptibility of CF pathogens and clinical isolates to cefiderocol and ceftazidime in CAMHB and M9+CAA. MIC values (µg/mL) are medians of at least three biological replicates. The ratio is the MIC in M9+CAA divided by the MIC in CAMHB. Bold indicates at least 2-fold difference between medium types.

		CFD			CAZ		
Species	Strain	CAMHB	M9+CAA	Ratio	CAMHB	M9+CAA	Ratio
A. xylosoxidans	ACH02	0.25	0.00781	**0.03125**	2	2	1
	ACH03	0.125	0.03125	**0.25**	1	1	1
S. maltophilia	D457	0.125	0.25	**2**	1	1	1
	K279a	0.125	0.125	1	2	4	**2**
P. aeruginosa	PAO1	1	0.0078	**0.0078**	1	1	1
	PA14	1	0.0078	**0.0078**	2	2	1
B. cenocepacia	110041	0.0625	0.5	**8**	4	2	**0.5**
	BC7	>4	>4	N/A	16	16	1
	K56-2	0.125	1	**4**	32	32	1
	VC10414	0.125	0.03125	**0.25**	8	8	1
	VC14488	0.25	4	**16**	4	8	**2**
	VC14543	0.125	1	**8**	8	4	**0.5**
	VC14761	0.125	0.125	1	4	8	**2**
B. dolosa	CEP021	0.5	>4	**>8**	2	2	1
B. multivorans	130034	0.125	0.5	**4**	8	8	1
	C5393	0.25	0.5	**2**	8	8	1
	VC15535	0.5	>8	**>16**	32	32	1
	VC19694	1	4	**4**	4	4	1
	VC9825	0.25	2	**8**	2	4	**2**
	ATCC17616	4	4	1	8	8	1
B. vietnamiensis	CEP40	0.125	2	**16**	4	4	1
	VC11431	1	4	**4**	32	32	1
	VC18984	>8	>8	N/A	16	8	**0.5**
B. thailandensis	E264	0.125	0.0625	**0.5**	8	4	**0.5**

Supplementary Table 6. Whole panel of CRISPRi mutants in genes associated with iron uptake from Figure 8 and their susceptibility to cefiderocol and ceftazidime in CAMHB and M9+CAA. MIC values (µg/mL) presented are medians of at least four biological replicates in the presence of 0.5% rhamnose. Bold indicates at least 2-fold difference from the NTC. Gene names are assigned by homology, if known, or else the locus tag is given (preceded by K562_).

Gene Function	CRISPRi Target	CFD		CAZ	
		CAMHB	M9+CAA	CAMHB	M9+CAA
N/A	NTC	0.125	1	32	32
TBDR (Outer membrane protein)	*piuA* (RS21350)	**0.5**	**4**	32	32
	RS00600	0.125	1	32	32
	RS07175	0.125	1	32	32
	RS09770	0.125	1	32	32
	RS09805	0.125	1	32	32
	cntO (RS10150)*	**0.25**	**2**	**8**	**16**
	orbA (RS10200)	0.125	1	32	32
	fecA (RS11910)	0.125	1	32	32
	RS12040	0.125	1	32	32
	RS14405	0.125	1	32	32
	RS19650	0.125	1	32	32
	RS19695	0.125	1	32	32
	RS20035	0.125	1	32	32
	RS20755	0.125	1	32	32
	RS21965	0.125	1	32	32
	RS25090	0.125	1	32	32
	btuB (RS25215)	0.125	1	32	32
	RS27300	0.125	1	32	32
	fptA (RS28410)	0.125	1	32	32
	RS29155	0.125	1	32	32
	RS29535	0.125	1	32	**16**
	hmuR (RS30495)	**0.5**	1	32	32
	orbA2 (RS33275)	0.125	1	32	32
	RS33410	0.125	1	**16**	**16**
Regulator	*fur* (RS16355)	**0.03125**	**0.125**	**8**	**4**
Energy transduction to TBDRs	*tonB1* (RS20010)**	**0.0625**	**0.25**	32	**8**
	tonB2 (RS09735)	0.125	1	32	32
	tonB3 (RS07120)	**1**	**2**	32	32
	tonB4 (RS14515)	0.125	1	32	**16**

* K562_RS10150 was assigned as a strong homologue to *P. aeruginosa* PAO1 *cntO* but failed reciprocal best-hit BLAST.

** *tonB* paralogues are assigned based on similarity to the *P. aeruginosa* PAO1 *tonB1*

Supplementary Table 7. Bacterial strains and mutants used in this work.

Strain	Features	Source
Achromobacter xylosoxidans ACH02	Canadian CF isolate; multidrug resistant	H. Adam (Shared Health MB)
A. xylosoxidans ACH03	Canadian CF isolate; multidrug resistant	H. Adam (Shared Health MB)
A. xylosoxidans ACH09	Canadian CF isolate; multidrug resistant	H. Adam (Shared Health MB)
B. cenocepacia 110041	Canadian CF isolate	National Microbiology Lab, MB
B. cenocepacia K56-2	ET12 lineage CF clinical isolate from Toronto	[1]
B. cenocepacia K56-2::dCas9	Derived from K56-2; pAH-CTX1rhadCas9 integrated at *attB* site; clean deletion of plasmid accessory genes	[2]
B. cenocepacia BC7	Canadian CF isolate; epidemic lineage	CBCCRRR
B. cenocepacia VC10414	Canadian CF isolate	CBCCRRR
B. cenocepacia VC14488	Canadian CF isolate	CBCCRRR
B. cenocepacia VC14543	Canadian CF isolate	CBCCRRR
B. cenocepacia VC14761	Canadian CF isolate	CBCCRRR
B. cenocepacia 110041	Canadian CF isolate	National Microbiology Lab, Winnipeg, Canada
B. cenocepacia F4200989	CF isolate from SickKids Hospital, Toronto, Canada	Dr. Valerie Waters
B. cenocepacia G1181568-1	CF isolate from SickKids Hospital, Toronto, Canada	Dr. Valerie Waters
B. cenocepacia F3071909	CF isolate from SickKids Hospital, Toronto, Canada	Dr. Valerie Waters
B. cenocepacia F9141525	CF isolate from SickKids Hospital, Toronto, Canada	Dr. Valerie Waters
B. cenocepacia 130723	Respiratory isolate from Edmonton, Canada	Canadian Antimicrobial Resistance Alliance
B. cenocepacia 134991	Respiratory isolate from Halifax, Canada	Canadian Antimicrobial Resistance Alliance
B. cepacia ATCC 25416	Onion pathogen, reference strain	ATCC
B. contaminans 21NG595759	Canadian CF isolate	H. Adam (Shared Health MB)
B. contaminans 21NG609698	Canadian CF isolate	H. Adam (Shared Health MB)
B. contaminans FFH2055	Cystic fibrosis clinical isolate from Argentina	[3]
B. contaminans LMG23361	Veterinary isolate	BCCM
B. dolosa CEP021	Cystic fibrosis isolate	Dr. David Speert

B. dolosa AU0645	CF clinical isolate	Dr. Sarah Kennedy/Dr. Valerie Waters
B. dolosa LM018941	CF clinical isolate	Dr. Sarah Kennedy/Dr. Valerie Waters
B. dolosa AU2167	CF clinical isolate	Dr. Sarah Kennedy/Dr. Valerie Waters
B. dolosa AU6940	CF clinical isolate	Dr. Sarah Kennedy/Dr. Valerie Waters
B. gladioli VC14812	Canadian CF isolate	CBCCRRR
B. gladioli VC19233	Canadian CF isolate	CBCCRRR
B. gladioli 113611	Respiratory isolate from Saskatoon, Canada	Canadian Antimicrobial Resistance Alliance
B. gladioli 132208	Respiratory isolate from Montréal, Canada	Canadian Antimicrobial Resistance Alliance
B. multivorans 130034	Canadian CF isolate	National Microbiology Lab, MB
B. multivorans 21NH942533	Canadian CF isolate	H. Adam (Shared Health MB)
B. multivorans 21NJ503211	Canadian CF isolate	H. Adam (Shared Health MB)
B. mutlivorans ATCC 17616	Soil isolate	ATCC
B. multivorans VC15535	Canadian CF isolate	CBCCRRR
B. multivorans VC15555	Canadian CF isolate	CBCCRRR
B. multivorans VC19694	Canadian CF isolate	CBCCRRR
B. multivorans VC9825	Canadian CF isolate	CBCCRRR
B. multivorans 131077	Respiratory isolate from Toronto, Canada	Canadian Antimicrobial Resistance Alliance
B. multivorans 134955	Respiratory isolate from Edmonton, Canada	Canadian Antimicrobial Resistance Alliance
B. multivorans F9091687	CF clinical isolate	Dr. Sarah Kennedy/Dr. Valerie Waters
B. multivorans LM013010	CF clinical isolate	Dr. Sarah Kennedy/Dr. Valerie Waters

B. multivorans V1120157	CF clinical isolate	Dr. Sarah Kennedy/Dr. Valerie Waters
B. multivorans C5393	CF clinical isolate	Dr. David Speert
B. thailandensis E264	Rice-field soil sample in Thailand	DSMZ
B. vietnamiensis CEP40	American cystic fibrosis isolate	[4]
B. vietnamiensis VC11431	Canadian cystic fibrosis isolate	CBCCRRR
B. vietnamiensis VC18984	Canadian cystic fibrosis isolate	CBCCRRR
E. coli DH5α	F$^-$ Φ80*lac*ZΔM15 Δ(*lac*ZYA*arg*F) U169 *rec*A1 *end*A1 *hsd*R17 (rK$^-$, mK$^+$) *pho*A *sup*E44 λ$^-$ *thi*-1 *gyr*A96 *rel*A1	Invitrogen
E. coli MM294	F− *φ*80 *lacZ*ΔM15 *endA1 recA1 hsdR17* (rH− mK+) *supE44 thi-1* Δ*gyrA96* (*ΔlacZYA-argF*)U169 *relA1*	[5]
E. coli SY327	F$^-$ *araD* Δ(*lac-proAB*) *argE*(Am) *recA56* Rifr *nalA* λ*pir*	[6]
Pseudomonas. aeruginosa 20NB681421	Canadian cystic fibrosis isolate	H. Adam (Shared Health MB)
P. aeruginosa 21NE72623	Canadian cystic fibrosis isolate	H. Adam (Shared Health MB)
P. aeruginosa PAO1	Lab strain from burn wound	Dr. Ayush Kumar
P. aeruginosa 70	CF clinical isolate from Montréal, Canada	Dr. Valerie Waters
P. aeruginosa 71	CF clinical isolate from Montréal, Canada	Dr. Valerie Waters
P. aeruginosa 74	CF clinical isolate from Montréal, Canada	Dr. Valerie Waters
Stenotrophomonas maltophilia 20NC580771	Canadian CF isolate	H. Adam (Shared Health MB)
S. maltophilia 21NE928511	Canadian CF isolate	H. Adam (Shared Health MB)
S. maltophilia D457	Non-CF respiratory isolate from Spain	ATCC
S. maltophilia K279a	Bloodstream isolate from UK; multidrug resistant	ATCC
S. maltophilia 03 T20510	CF isolate from SickKids Hospital, Toronto, Canada	Dr. Valerie Waters
S. maltophilia 04 T22674	CF isolate from SickKids Hospital, Toronto, Canada	Dr. Valerie Waters
S. maltophilia 05 41846	CF isolate from SickKids Hospital, Toronto, Canada	Dr. Valerie Waters

Supplementary Table 8. Plasmids used in this work.

Plasmid	Features	Source
pDAI-SceI-*sacB*	pDAI-SceI expressing *sacB*	[7,8]
pGPI-SceI	ori_{R6K} Tmp^r mob^+ carries I-SceI cut site	[9]
pSCrhaBoutgfp	pTnMod-OTp′, *rhaR rhaS* P_{rhaB} *e-gfp*	[10]
pRBrhaBout	pSCrhaboutgfp derivative<br>ori_{R6K} *dhfr rhaR rhaS* P*rhaB* (also called pRB-rham)	[11]
pKS1	pRBrhaBout with *Nco*I and *Bam*HI restriction sites inserted after *dhfr*	This study
pRBrha-Barcode	Pool of pKS1 with the random barcode ligated in the *Nco*I and *Bam*HI sites	This study
pRK2013	ori_{colE1} RK2 derivative Kan^r mob^+ tra^+	[12]
pSCrhaB2-plus	pSCrhaB2 with $rhaI_1$-$rhaI_1$ permutation of *rhaS* binding sites upstream of P_{rhaBAD} and bacteriophage T7 *gene 10* stem loop inserted upstream of native *rhaBAD* 5′ UTR	[13]
pSCB2-sgRNAv2	Template for pgRNA created by inverse PCR; derived from pSCrhaB2-sgRNA by inverse<br>PCR to remove *rhaS, rhaR,* and<br>P_{rhaB}	[2]
pgRNA-non-target	Derived from pSCB2-sgRNA; random 20 nt sequence added as gRNA binding region by inverse PCR	[2]
p*hldD*+	pSCrhaB2plus with K56-2 *hldD* gene (K562_RS14060) cloned between *Nde*I and *Xba*I sites	This study
p*waaL*+	pSCrhaB2plus with K56-2 *waaL* gene (K562_RS06530) cloned between *Nde*I and *Xba*I sites	This study
p*wbiI*+	pSCrhaB2plus with K56-2 *wbiI* gene (K562_RS15035) cloned between *Nde*I and *Xba*I sites	This study
p*wzm-wzt*+	pSCrhaB2plus with K56-2 *wzm* and *wzt* genes (K562_RS15080 and K562_RS15085) cloned between *Nde*I and *Xba*I sites	This study
pGPI-SceI-*hldD*	pGPI-SceI with a fusion of 360 bp regions immediately upstream and downstream of *hldD* cloned into *Xba*I and *Xma*I sites. Enables deletion of the 3’ 796/993 bp of *hldD*.	This study
pGPI-SceI-*waaL*	pGPI-SceI with a fusion of 360 bp regions immediately upstream and downstream of *waaL* cloned into *Xba*I and *Xma*I sites. Enables deletion of the 5’ 785/1230 bp of *waaL*.	This study
pgRNA-32470-1	pSCB2-sgRNAv2 expressing sgRNA targeting K562_RS32470	This study

pgRNA-32470-2	pSCB2-sgRNAv2 expressing sgRNA targeting K562_RS32470	This study
pgRNA-*ampC*1	pSCB2-sgRNAv2 expressing sgRNA targeting *ampC*	This study
pgRNA-*ampC*2	pSCB2-sgRNAv2 expressing sgRNA targeting *ampC*	This study
pgRNA-*ampD*1	pSCB2-sgRNAv2 expressing sgRNA targeting *ampD*	This study
pgRNA-*ampD*2	pSCB2-sgRNAv2 expressing sgRNA targeting *ampD*	This study
pgRNA-*ampG*1	pSCB2-sgRNAv2 expressing sgRNA targeting *ampG*	This study
pgRNA-*ampG*2	pSCB2-sgRNAv2 expressing sgRNA targeting *ampG*	This study
pgRNA-*bacA*1	pSCB2-sgRNAv2 expressing sgRNA targeting *bacA*	This study
pgRNA-*bacA*2	pSCB2-sgRNAv2 expressing sgRNA targeting *bacA*	This study
pgRNA-*hldD*1	pSCB2-sgRNAv2 expressing sgRNA targeting *hldD*	This study
pgRNA-*hldD*2	pSCB2-sgRNAv2 expressing sgRNA targeting *hldD*	This study
pgRNA-*ispDF*1	pSCB2-sgRNAv2 expressing sgRNA targeting *ispDF* operon	This study
pgRNA-*ispDF*2	pSCB2-sgRNAv2 expressing sgRNA targeting *ispDF* operon	This study
pgRNA-*mlaFEDvacJ*1	pSCB2-sgRNAv2 expressing sgRNA targeting the *mlaFEDvacJ* operon	This study
pgRNA-*mlaFEDvacJ*2	pSCB2-sgRNAv2 expressing sgRNA targeting the *mlaFEDvacJ* operon	This study
pgRNA-*mlaCB*1	pSCB2-sgRNAv2 expressing sgRNA targeting the *mlaCB* operon	This study
pgRNA-*mlaCB*2	pSCB2-sgRNAv2 expressing sgRNA targeting the *mlaCB* operon	This study
pgRNA-*ogcABEI*1	pSCB2-sgRNAv2 expressing sgRNA targeting *ogcABEI* operon	This study
pgRNA-*ogcABEI*2	pSCB2-sgRNAv2 expressing sgRNA targeting *ogcABEI* operon	This study
pgRNA-*penB*1	pSCB2-sgRNAv2 expressing sgRNA targeting *penB*	This study
pgRNA-*penB*2	pSCB2-sgRNAv2 expressing sgRNA targeting *penB*	This study
pgRNA-*penR*1	pSCB2-sgRNAv2 expressing sgRNA targeting *penR*	This study
pgRNA-*penR*2	pSCB2-sgRNAv2 expressing sgRNA targeting *penR*	This study
pgRNA-*pglL*1	pSCB2-sgRNAv2 expressing sgRNA targeting *pglL*	This study
pgRNA-*pglL*2	pSCB2-sgRNAv2 expressing sgRNA targeting *pglL*	This study
pgRNA-*piuA*1	pSCB2-sgRNAv2 expressing sgRNA targeting *piuA*	This study
pgRNA-*piuA*2	pSCB2-sgRNAv2 expressing sgRNA targeting *piuA*	This study
pgRNA-*uppS*1	pSCB2-sgRNAv2 expressing sgRNA targeting *uppS*	This study
pgRNA-*uppS*2	pSCB2-sgRNAv2 expressing sgRNA targeting *uppS*	This study
pgRNA-*vio-wbx*1	pSCB2-sgRNAv2 expressing sgRNA targeting *vio-wbx* operon	This study

pgRNA-*vio-wbx*2	pSCB2-sgRNAv2 expressing sgRNA targeting *vio-wbx* operon	This study
pgRNA-*waaL*1	pSCB2-sgRNAv2 expressing sgRNA targeting *waaL*	This study
pgRNA-*waaL*2	pSCB2-sgRNAv2 expressing sgRNA targeting *waaL*	This study
pgRNA-*wabR-P*1	pSCB2-sgRNAv2 expressing sgRNA targeting *wabR-P* operon	This study
pgRNA-*wabR-P*2	pSCB2-sgRNAv2 expressing sgRNA targeting *wabR-P* operon	This study
pgRNA-*wbiFGHI*1	pSCB2-sgRNAv2 expressing sgRNA targeting *wbiFGHI* operon	This study
pgRNA-*wbiFGHI*2	pSCB2-sgRNAv2 expressing sgRNA targeting *wbiFGHI* operon	This study
pgRNA-*wzm-wzt*1	pSCB2-sgRNAv2 expressing sgRNA targeting *wzm-wzt* operon	This study
pgRNA-*wzm-wzt*2	pSCB2-sgRNAv2 expressing sgRNA targeting *wzm-wzt* operon	This study
pgRNA-00600-1	pSCB2-sgRNAv2 expressing sgRNA targeting K562_RS00600	This study
pgRNA-00600-2	pSCB2-sgRNAv2 expressing sgRNA targeting K562_RS00600	This study
pgRNA-07175-1	pSCB2-sgRNAv2 expressing sgRNA targeting K562_RS07175	This study
pgRNA-07175-2	pSCB2-sgRNAv2 expressing sgRNA targeting K562_RS07175	This study
pgRNA-09770-1	pSCB2-sgRNAv2 expressing sgRNA targeting K562_RS09770	This study
pgRNA-09770-2	pSCB2-sgRNAv2 expressing sgRNA targeting K562_RS09770	This study
pgRNA-09805-1	pSCB2-sgRNAv2 expressing sgRNA targeting K562_RS09805	This study
pgRNA-09805-2	pSCB2-sgRNAv2 expressing sgRNA targeting K562_RS09805	This study
pgRNA-10150-1	pSCB2-sgRNAv2 expressing sgRNA targeting K562_RS10150	This study
pgRNA-10150-2	pSCB2-sgRNAv2 expressing sgRNA targeting K562_RS0150	This study
pgRNA-10200-1	pSCB2-sgRNAv2 expressing sgRNA targeting K562_RS10200	This study
pgRNA-10200-2	pSCB2-sgRNAv2 expressing sgRNA targeting K562_RS10200	This study

pgRNA-11910-1	pSCB2-sgRNAv2 expressing sgRNA targeting K562_RS11910	This study
pgRNA-11910-2	pSCB2-sgRNAv2 expressing sgRNA targeting K562_RS11910	This study
pgRNA-12040-1	pSCB2-sgRNAv2 expressing sgRNA targeting K562_RS12040	This study
pgRNA-12040-2	pSCB2-sgRNAv2 expressing sgRNA targeting K562_RS12040	This study
pgRNA-14405-1	pSCB2-sgRNAv2 expressing sgRNA targeting K562_RS14405	This study
pgRNA-14405-2	pSCB2-sgRNAv2 expressing sgRNA targeting K562_RS14405	This study
pgRNA-19650-1	pSCB2-sgRNAv2 expressing sgRNA targeting K562_RS19650	This study
pgRNA-19650-2	pSCB2-sgRNAv2 expressing sgRNA targeting K562_RS19650	This study
pgRNA-19695-1	pSCB2-sgRNAv2 expressing sgRNA targeting K562_RS19695	This study
pgRNA-19695-2	pSCB2-sgRNAv2 expressing sgRNA targeting K562_RS19695	This study
pgRNA-20035-1	pSCB2-sgRNAv2 expressing sgRNA targeting K562_RS20035	This study
pgRNA-20035-2	pSCB2-sgRNAv2 expressing sgRNA targeting K562_RS20035	This study
pgRNA-20755-1	pSCB2-sgRNAv2 expressing sgRNA targeting K562_RS20755	This study
pgRNA-20755-2	pSCB2-sgRNAv2 expressing sgRNA targeting K562_RS20755	This study
pgRNA-21965-1	pSCB2-sgRNAv2 expressing sgRNA targeting K562_RS21965	This study
pgRNA-21965-2	pSCB2-sgRNAv2 expressing sgRNA targeting K562_RS21965	This study
pgRNA-25090-1	pSCB2-sgRNAv2 expressing sgRNA targeting K562_RS25090	This study
pgRNA-25090-2	pSCB2-sgRNAv2 expressing sgRNA targeting K562_RS25090	This study
pgRNA-25215-1	pSCB2-sgRNAv2 expressing sgRNA targeting K562_RS25215	This study
pgRNA-25215-2	pSCB2-sgRNAv2 expressing sgRNA targeting K562_RS25215	This study

pgRNA-27300-1	pSCB2-sgRNAv2 expressing sgRNA targeting K562_RS27300	This study
pgRNA-27300-2	pSCB2-sgRNAv2 expressing sgRNA targeting K562_RS27300	This study
pgRNA-28410-1	pSCB2-sgRNAv2 expressing sgRNA targeting K562_RS28410	This study
pgRNA-28410-2	pSCB2-sgRNAv2 expressing sgRNA targeting K562_RS28410	This study
pgRNA-29155-1	pSCB2-sgRNAv2 expressing sgRNA targeting K562_RS29155	This study
pgRNA-29155-2	pSCB2-sgRNAv2 expressing sgRNA targeting K562_RS29155	This study
pgRNA-29535-1	pSCB2-sgRNAv2 expressing sgRNA targeting K562_RS29535	This study
pgRNA-29535-2	pSCB2-sgRNAv2 expressing sgRNA targeting K562_RS29535	This study
pgRNA-30495-1	pSCB2-sgRNAv2 expressing sgRNA targeting K562_RS30495	This study
pgRNA-30495-2	pSCB2-sgRNAv2 expressing sgRNA targeting K562_RS30495	This study
pgRNA-33275-1	pSCB2-sgRNAv2 expressing sgRNA targeting K562_RS33275	This study
pgRNA-33275-2	pSCB2-sgRNAv2 expressing sgRNA targeting K562_RS33275	This study
pgRNA-33410-1	pSCB2-sgRNAv2 expressing sgRNA targeting K562_RS33410	This study
pgRNA-33410-2	pSCB2-sgRNAv2 expressing sgRNA targeting K562_RS33410	This study
pgRNA-16355-1	pSCB2-sgRNAv2 expressing sgRNA targeting K562_RS16355	This study
pgRNA-16355-2	pSCB2-sgRNAv2 expressing sgRNA targeting K562_RS16355	This study
pgRNA-20010-1	pSCB2-sgRNAv2 expressing sgRNA targeting K562_RS20010	This study
pgRNA-20010-2	pSCB2-sgRNAv2 expressing sgRNA targeting K562_RS20010	This study
pgRNA-09735-1	pSCB2-sgRNAv2 expressing sgRNA targeting K562_RS09735	This study
pgRNA-09735-2	pSCB2-sgRNAv2 expressing sgRNA targeting K562_RS09735	This study

pgRNA-07120-1	pSCB2-sgRNAv2 expressing sgRNA targeting K562_RS07120	This study
pgRNA-07120-2	pSCB2-sgRNAv2 expressing sgRNA targeting K562_RS07120	This study
pgRNA-14515-1	pSCB2-sgRNAv2 expressing sgRNA targeting K562_RS14515	This study
pgRNA-14515-2	pSCB2-sgRNAv2 expressing sgRNA targeting K562_RS14515	This study

Supplementary Table 9. Primers used in this work.

Primer	Sequence (restriction sites in lowercase)	Notes
681	CAAGCAGAAGACGGCATACGAGATTCGCCTTAGTCTCGTG G GCTCGGAGATGTGTATAAGAGACAG	Used for TnSeq circle
682	CACAAGTGCGGCCGCACTAGTCTAGATTTAAATTACCGTA GTGAGTTCTTCGTCCGAGCCAC	Collector probe for TnSeq-circle
683	P – CCGTAGTGAGTTCTTCGTCCGAGCCACTCGGAGATGTGTA T AAGAGACAGT	Half of adapter for TnSeq-circle
684	P – CTGTCTCTTATACACATCTCCGAGTGGCTCGGACGAAGAA C TCACTACGG	Half of adapter for TnSeq-circle
690	AATGATACGGCGACCACCGAGATCTACACTAGATCGCTCG TCGGCAGCGTCAGATGTGTATAAGAGACAGNNNNAATCT AGACTAGTGCGGCC	Used for TnSeq circle
715	CAAGCAGAAGACGGCATACGAGATCTAGTACGGTCTCGTG G GCTCGGAGATGTGTATAAGAGACAG	Used for TnSeq circle
717	CAAGCAGAAGACGGCATACGAGATGCTCAGGAGTCTCGT GG GCTCGGAGATGTGTATAAGAGACAG	Used for TnSeq circle
718	CAAGCAGAAGACGGCATACGAGATAGGAGTCCGTCTCGT GG GCTCGGAGATGTGTATAAGAGACAG	Used for TnSeq circle
719	AATGATACGGCGACCACCGAGATCTACACCTCTCTATTCG TCGGCAGCGTCAGATGTGTATAAGAGACAGNNNNAATCT AGACTAGTGCGGCC	Used for TnSeq circle
729	CAAGCAGAAGACGGCATACGAGATCATGCCTAGTCTCGTG G GCTCGGAGATGTGTATAAGAGACAG	Used for TnSeq circle
781	TAAGATggatccTCAGTTGGCTTCATCGCTAC	Colony PCR for pSCB2-sgRNA
847	TTCCTGTCAGTAACGAGAAGG	Colony PCR for plasmids based on pSCrhaB2; used with 1025 to create pSCB2-sgRNA
848	CCGCCAGGCAAATTCTGTTT	Reverse primer for colony PCR, all gRNAs
964	CACTTGTGTATAAGAGTCAG	Reverse primer for In-fusion construction of pKS1
965	CTTATACACAAGTGCccatggGTCTTCAACGAGGCAggatccGG CCGCACTAGTCTAGA	Forward primer for In-fusion construction of pKS1
972	ATAATACCATGGATGTCCACGAGGTCTCT	Used to amplify the barcode during pRBrha-Barcode construction

973	AATTAAGGATCCGTCGACCTGCAGCGTACG	Used to amplify the barcode during pRBrha-Barcode construction
1092	ACTAGTATTATACCTAGGACTGAGCTAGC	Reverse primer, all gRNAs
1093	GTTTTAGAGCTAGAAATAGCAAGTTAAAATAAGGC	Forward primer, control gRNA
1409	GGCTTATGTCAACTGGGTTCG	Forward primer for general colony PCR of sgRNA plasmids
1429	GGATCCGGCCGCACTAGTCTAGATTTAAATTACCGTAGTG AGTTCTTCGTCCGAGCCAC	Collector probe for RB-TnSeq-circle
2163	CAAGCAGAAGACGGCATACGAGATGCTTACGGACGTCTC GTGGGCTCGGAGATGTGTATAAGAGACAGGTCGACCTGCA GCGTACG	BarSeq primer with i7 index; UDP0089
2164	CAAGCAGAAGACGGCATACGAGATCGCTTGAAGTGTCTCG TGGGCTCGGAGATGTGTATAAGAGACAGGTCGACCTGCAG CGTACG	BarSeq primer with i7 index; UDP0090
2165	CAAGCAGAAGACGGCATACGAGATCGCCTTCTGAGTCTCG TGGGCTCGGAGATGTGTATAAGAGACAGGTCGACCTGCAG CGTACG	BarSeq primer with i7 index; UDP0091
2166	CAAGCAGAAGACGGCATACGAGATCTGGATATGTGTCTCG TGGGCTCGGAGATGTGTATAAGAGACAGGTCGACCTGCAG CGTACG	BarSeq primer with i7 index; UDP0092
2167	CAAGCAGAAGACGGCATACGAGATATACCAACGCGTCTC GTGGGCTCGGAGATGTGTATAAGAGACAGGTCGACCTGCA GCGTACG	BarSeq primer with i7 index; UDP0093
2168	CAAGCAGAAGACGGCATACGAGATCAATCTATGAGTCTCG TGGGCTCGGAGATGTGTATAAGAGACAGGTCGACCTGCAG CGTACG	BarSeq primer with i7 index; UDP0094
2169	CAAGCAGAAGACGGCATACGAGATGGTGGAATACGTCTC GTGGGCTCGGAGATGTGTATAAGAGACAGGTCGACCTGCA GCGTACG	BarSeq primer with i7 index; UDP0095
2170	CAAGCAGAAGACGGCATACGAGATTGGACGGAGGGTCTC GTGGGCTCGGAGATGTGTATAAGAGACAGGTCGACCTGCA GCGTACG	BarSeq primer with i7 index; UDP0096
2171	AATGATACGGCGACCACCGAGATCTACACCGTGTATCTTT CGTCGGCAGCGTCAGATGTGTATAAGAGACAGGATGTCCA CGAGGTCTCT	BarSeq primer with i5 index; UDP0089
2172	AATGATACGGCGACCACCGAGATCTACACGAACCATGAAT CGTCGGCAGCGTCAGATGTGTATAAGAGACAGGATGTCCA CGAGGTCTCT	BarSeq primer with i5 index; UDP0090
2173	AATGATACGGCGACCACCGAGATCTACACGGCCATCATAT CGTCGGCAGCGTCAGATGTGTATAAGAGACAGGATGTCCA CGAGGTCTCT	BarSeq primer with i5 index; UDP0091

2174	AATGATACGGCGACCACCGAGATCTACACACATACTTCCT CGTCGGCAGCGTCAGATGTGTATAAGAGACAGGATGTCCA CGAGGTCTCT	BarSeq primer with i5 index; UDP0092
2175	AATGATACGGCGACCACCGAGATCTACACTATGTGCAATT CGTCGGCAGCGTCAGATGTGTATAAGAGACAGGATGTCCA CGAGGTCTCT	BarSeq primer with i5 index; UDP0093
2176	AATGATACGGCGACCACCGAGATCTACACGATTAAGGTGT CGTCGGCAGCGTCAGATGTGTATAAGAGACAGGATGTCCA CGAGGTCTCT	BarSeq primer with i5 index; UDP0094
2177	AATGATACGGCGACCACCGAGATCTACACATGTAGACAAT CGTCGGCAGCGTCAGATGTGTATAAGAGACAGGATGTCCA CGAGGTCTCT	BarSeq primer with i5 index; UDP0095
2178	AATGATACGGCGACCACCGAGATCTACACCACATCGGTGT CGTCGGCAGCGTCAGATGTGTATAAGAGACAGGATGTCCA CGAGGTCTCT	BarSeq primer with i5 index; UDP0096
2208	CAAGCAGAAGACGGCATACGAGATAGACACATTAGTCTC GTGGGCTCGGAGATGTGTATAAGAGACAGGTCGACCTGCA GCGTACG	BarSeq primer with i7 index; UDP0065
2209	AATGATACGGCGACCACCGAGATCTACACGTAAGGCATAT CGTCGGCAGCGTCAGATGTGTATAAGAGACAGGATGTCCA CGAGGTCTCT	BarSeq primer with i5 index; UDP0065
2210	CAAGCAGAAGACGGCATACGAGATGCGTTGGTATGTCTCG TGGGCTCGGAGATGTGTATAAGAGACAGGTCGACCTGCAG CGTACG	BarSeq primer with i7 index; UDP0066
2211	AATGATACGGCGACCACCGAGATCTACACAATTGCTGCGT CGTCGGCAGCGTCAGATGTGTATAAGAGACAGGATGTCCA CGAGGTCTCT	BarSeq primer with i5 index; UDP0066
2212	CAAGCAGAAGACGGCATACGAGATAGCACATCCTGTCTCG TGGGCTCGGAGATGTGTATAAGAGACAGGTCGACCTGCAG CGTACG	BarSeq primer with i7 index; UDP0067
2213	AATGATACGGCGACCACCGAGATCTACACTTACAATTCCT CGTCGGCAGCGTCAGATGTGTATAAGAGACAGGATGTCCA CGAGGTCTCT	BarSeq primer with i5 index; UDP0067
2214	CAAGCAGAAGACGGCATACGAGATTTGTTCCGTGGTCTCG TGGGCTCGGAGATGTGTATAAGAGACAGGTCGACCTGCAG CGTACG	BarSeq primer with i7 index; UDP0068
2215	AATGATACGGCGACCACCGAGATCTACACAACCTAGCACT CGTCGGCAGCGTCAGATGTGTATAAGAGACAGGATGTCCA CGAGGTCTCT	BarSeq primer with i5 index; UDP0068
2216	CAAGCAGAAGACGGCATACGAGATAAGTACTCCAGTCTC GTGGGCTCGGAGATGTGTATAAGAGACAGGTCGACCTGCA GCGTACG	BarSeq primer with i7 index; UDP0069
2217	AATGATACGGCGACCACCGAGATCTACACTCTGTGTGGAT CGTCGGCAGCGTCAGATGTGTATAAGAGACAGGATGTCCA CGAGGTCTCT	BarSeq primer with i5 index; UDP0069

2218	CAAGCAGAAGACGGCATACGAGATACGTCAATACGTCTCGTGGGCTCGGAGATGTGTATAAGAGACAGGTCGACCTGCAGCGTACG	BarSeq primer with i7 index; UDP0070
2219	AATGATACGGCGACCACCGAGATCTACACGGAATTCCAATCGTCGGCAGCGTCAGATGTGTATAAGAGACAGGATGTCCACGAGGTCTCT	BarSeq primer with i5 index; UDP0070
2220	CAAGCAGAAGACGGCATACGAGATGGTGTACAAGGTCTCGTGGGCTCGGAGATGTGTATAAGAGACAGGTCGACCTGCAGCGTACG	BarSeq primer with i7 index; UDP0071
2221	AATGATACGGCGACCACCGAGATCTACACAAGCGCGCTTTCGTCGGCAGCGTCAGATGTGTATAAGAGACAGGATGTCCACGAGGTCTCT	BarSeq primer with i5 index; UDP0071
2222	CAAGCAGAAGACGGCATACGAGATCCACCTGTGTGTCTCGTGGGCTCGGAGATGTGTATAAGAGACAGGTCGACCTGCAGCGTACG	BarSeq primer with i7 index; UDP0072
2223	AATGATACGGCGACCACCGAGATCTACACTGAGCGTTGTTCGTCGGCAGCGTCAGATGTGTATAAGAGACAGGATGTCCACGAGGTCTCT	BarSeq primer with i5 index; UDP0072
2224	CAAGCAGAAGACGGCATACGAGATGTTCCGCAGGGTCTCGTGGGCTCGGAGATGTGTATAAGAGACAGGTCGACCTGCAGCGTACG	BarSeq primer with i7 index; UDP0073
2225	AATGATACGGCGACCACCGAGATCTACACATCATAGGCTTCGTCGGCAGCGTCAGATGTGTATAAGAGACAGGATGTCCACGAGGTCTCT	BarSeq primer with i5 index; UDP0073
2226	CAAGCAGAAGACGGCATACGAGATACCTTATGAAGTCTCGTGGGCTCGGAGATGTGTATAAGAGACAGGTCGACCTGCAGCGTACG	BarSeq primer with i7 index; UDP0074
2227	AATGATACGGCGACCACCGAGATCTACACTGTTAGAAGGTCGTCGGCAGCGTCAGATGTGTATAAGAGACAGGATGTCCACGAGGTCTCT	BarSeq primer with i5 index; UDP0074
2228	CAAGCAGAAGACGGCATACGAGATCGCTGCAGAGGTCTCGTGGGCTCGGAGATGTGTATAAGAGACAGGTCGACCTGCAGCGTACG	BarSeq primer with i7 index; UDP0075
2229	AATGATACGGCGACCACCGAGATCTACACGATGGATGTATCGTCGGCAGCGTCAGATGTGTATAAGAGACAGGATGTCCACGAGGTCTCT	BarSeq primer with i5 index; UDP0075
2230	CAAGCAGAAGACGGCATACGAGATGTAGAGTCAGGTCTCGTGGGCTCGGAGATGTGTATAAGAGACAGGTCGACCTGCAGCGTACG	BarSeq primer with i7 index; UDP0076
2231	AATGATACGGCGACCACCGAGATCTACACACGGCCGTCATCGTCGGCAGCGTCAGATGTGTATAAGAGACAGGATGTCCACGAGGTCTCT	BarSeq primer with i5 index; UDP0076
2232	CAAGCAGAAGACGGCATACGAGATGGATACCAGAGTCTCGTGGGCTCGGAGATGTGTATAAGAGACAGGTCGACCTGCAGCGTACG	BarSeq primer with i7 index; UDP0077

2233	AATGATACGGCGACCACCGAGATCTACACCGTTGCTTACTCGTCGGCAGCGTCAGATGTGTATAAGAGACAGGATGTCCACGAGGTCTCT	BarSeq primer with i5 index; UDP0077
2234	CAAGCAGAAGACGGCATACGAGATCGCACTAATGGTCTCGTGGGCTCGGAGATGTGTATAAGAGACAGGTCGACCTGCAGCGTACG	BarSeq primer with i7 index; UDP0078
2235	AATGATACGGCGACCACCGAGATCTACACTGACTACATATCGTCGGCAGCGTCAGATGTGTATAAGAGACAGGATGTCCACGAGGTCTCT	BarSeq primer with i5 index; UDP0078
2236	CAAGCAGAAGACGGCATACGAGATTCCTGACCGTGTCTCGTGGGCTCGGAGATGTGTATAAGAGACAGGTCGACCTGCAGCGTACG	BarSeq primer with i7 index; UDP0079
2237	AATGATACGGCGACCACCGAGATCTACACCGGCCTCGTTTCGTCGGCAGCGTCAGATGTGTATAAGAGACAGGATGTCCACGAGGTCTCT	BarSeq primer with i5 index; UDP0079
2238	CAAGCAGAAGACGGCATACGAGATCTGGCTTGCCGTCTCGTGGGCTCGGAGATGTGTATAAGAGACAGGTCGACCTGCAGCGTACG	BarSeq primer with i7 index; UDP0080
2239	AATGATACGGCGACCACCGAGATCTACACCAAGCATCCGTCGTCGGCAGCGTCAGATGTGTATAAGAGACAGGATGTCCACGAGGTCTCT	BarSeq primer with i5 index; UDP0080
2240	CAAGCAGAAGACGGCATACGAGATACCAGCGACAGTCTCGTGGGCTCGGAGATGTGTATAAGAGACAGGTCGACCTGCAGCGTACG	BarSeq primer with i7 index; UDP0081
2241	AATGATACGGCGACCACCGAGATCTACACTCGTCTGACTTCGTCGGCAGCGTCAGATGTGTATAAGAGACAGGATGTCCACGAGGTCTCT	BarSeq primer with i5 index; UDP0081
2242	CAAGCAGAAGACGGCATACGAGATTTGTAACGGTGTCTCGTGGGCTCGGAGATGTGTATAAGAGACAGGTCGACCTGCAGCGTACG	BarSeq primer with i7 index; UDP0082
2243	AATGATACGGCGACCACCGAGATCTACACCTCATAGCGATCGTCGGCAGCGTCAGATGTGTATAAGAGACAGGATGTCCACGAGGTCTCT	BarSeq primer with i5 index; UDP0082
2244	CAAGCAGAAGACGGCATACGAGATGTAAGGCATAGTCTCGTGGGCTCGGAGATGTGTATAAGAGACAGGTCGACCTGCAGCGTACG	BarSeq primer with i7 index; UDP0083
2245	AATGATACGGCGACCACCGAGATCTACACAGACACATTATCGTCGGCAGCGTCAGATGTGTATAAGAGACAGGATGTCCACGAGGTCTCT	BarSeq primer with i5 index; UDP0083
2246	CAAGCAGAAGACGGCATACGAGATGTCCACTTGTGTCTCGTGGGCTCGGAGATGTGTATAAGAGACAGGTCGACCTGCAGCGTACG	BarSeq primer with i7 index; UDP0084
2247	AATGATACGGCGACCACCGAGATCTACACGCGCGATGTTTCGTCGGCAGCGTCAGATGTGTATAAGAGACAGGATGTCCACGAGGTCTCT	BarSeq primer with i5 index; UDP0084

2248	CAAGCAGAAGACGGCATACGAGATTTAGGTACCAGTCTCGTGGGCTCGGAGATGTGTATAAGAGACAGGTCGACCTGCAGCGTACG	BarSeq primer with i7 index; UDP0085
2249	AATGATACGGCGACCACCGAGATCTACACCATGAGTACTTCGTCGGCAGCGTCAGATGTGTATAAGAGACAGGATGTCCACGAGGTCTCT	BarSeq primer with i5 index; UDP0085
2250	CAAGCAGAAGACGGCATACGAGATGGAATTCCAAGTCTCGTGGGCTCGGAGATGTGTATAAGAGACAGGTCGACCTGCAGCGTACG	BarSeq primer with i7 index; UDP0086
2251	AATGATACGGCGACCACCGAGATCTACACACGTCAATACTCGTCGGCAGCGTCAGATGTGTATAAGAGACAGGATGTCCACGAGGTCTCT	BarSeq primer with i5 index; UDP0086
2252	CAAGCAGAAGACGGCATACGAGATCATGTAGAGGGTCTCGTGGGCTCGGAGATGTGTATAAGAGACAGGTCGACCTGCAGCGTACG	BarSeq primer with i7 index; UDP0087
2253	AATGATACGGCGACCACCGAGATCTACACGATACCTCCTTCGTCGGCAGCGTCAGATGTGTATAAGAGACAGGATGTCCACGAGGTCTCT	BarSeq primer with i5 index; UDP0087
2254	CAAGCAGAAGACGGCATACGAGATTACACGCTCCGTCTCGTGGGCTCGGAGATGTGTATAAGAGACAGGTCGACCTGCAGCGTACG	BarSeq primer with i7 index; UDP0088
2255	AATGATACGGCGACCACCGAGATCTACACATCCGTAAGTTCGTCGGCAGCGTCAGATGTGTATAAGAGACAGGATGTCCACGAGGTCTCT	BarSeq primer with i5 index; UDP0088
2421	GAGCGGGGTCGGGCAAGCAGGTTTTAGAGCTAGAAATAGCAAGTTAAAATAAGGC	Primer containing sgRNA to target *ogcABEI* operon
2422	TCGTCGTGCCCGTTGTAGGCGTTTTAGAGCTAGAAATAGCAAGTTAAAATAAGGC	Primer containing sgRNA to target *ogcABEI* operon
2445	TGCGGCGACCAGCAGGGTCGGTTTTAGAGCTAGAAATAGCAAGTTAAAATAAGGC	Primer containing sgRNA to target *ampC*
2446	TCTGGCGCGTGACGGTGTCGGTTTTAGAGCTAGAAATAGCAAGTTAAAATAAGGC	Primer containing sgRNA to target *ampC*
2453	GGCCTCGTCTTGAACTGGGCGTTTTAGAGCTAGAAATAGCAAGTTAAAATAAGGC	Primer containing sgRNA to target *piuA*
2454	GTTTCGGCGGAGGCGAGCAGGTTTTAGAGCTAGAAATAGCAAGTTAAAATAAGGC	Primer containing sgRNA to target *piuA*
2457	CGTCGCGGCGGCCAGCAACAGTTTTAGAGCTAGAAATAGCAAGTTAAAATAAGGC	Primer containing sgRNA to target *penB*
2458	GCGGTGGCGGTGAGAACGAGGTTTTAGAGCTAGAAATAGCAAGTTAAAATAAGGC	Primer containing sgRNA to target *penB*
2459	CGTCACGCTCAGCTCGAGGCGTTTTAGAGCTAGAAATAGCAAGTTAAAATAAGGC	Primer containing sgRNA to target *penR*
2460	CTGAACGCGTCGCTCAGCACGTTTTAGAGCTAGAAATAGCAAGTTAAAATAAGGC	Primer containing sgRNA to target *penR*
2461	GGAATCAGGGCGAAAAGTCGGTTTTAGAGCTAGAAATAGCAAGTTAAAATAAGGC	Primer containing sgRNA to target *ispDF* operon

2462	CGTGCCGGCACAGGGAATCAGTTTTAGAGCTAGAAATAGC AAGTTAAAATAAGGC	Primer containing sgRNA to target *ispDF* operon
2467	GCCGACGTCAGGCACGCGAAGTTTTAGAGCTAGAAATAGC AAGTTAAAATAAGGC	Primer containing sgRNA to target *uppS* operon
2468	ATGATGATCGCGATGTGACGGTTTTAGAGCTAGAAATAGC AAGTTAAAATAAGGC	Primer containing sgRNA to target *uppS* operon
2469	GATGTTCGCGCCGATAAAACGTTTTAGAGCTAGAAATAGC AAGTTAAAATAAGGC	Primer containing sgRNA to target *hldD*
2470	ATTGCGAAACTTGTCTGCGCGTTTTAGAGCTAGAAATAGC AAGTTAAAATAAGGC	Primer containing sgRNA to target *hldD*
2477	TGTTTCGTCGACGGAATGCGGTTTTAGAGCTAGAAATAGC AAGTTAAAATAAGGC	Primer containing sgRNA to target *wbiFGHI*
2478	GCGGAGACTGGAGAGCGTCCGTTTTAGAGCTAGAAATAGC AAGTTAAAATAAGGC	Primer containing sgRNA to target *wbiFGHI* operon
2479	ATCGGCTTGTCGAATACGTCGTTTTAGAGCTAGAAATAGC AAGTTAAAATAAGGC	Primer containing sgRNA to target *vio-wbx* operon
2480	ATTGGCTTGGTCACATAGATGTTTTAGAGCTAGAAATAGC AAGTTAAAATAAGGC	Primer containing sgRNA to target *vio-wbx* operon
2481	TGTGGATACGTCCGGGGAAGGTTTTAGAGCTAGAAATAGC AAGTTAAAATAAGGC	Primer containing sgRNA to target *wabR-P* operon
2482	AGCGGCTTGAACAGCAATCTGTTTTAGAGCTAGAAATAGC AAGTTAAAATAAGGC	Primer containing sgRNA to target *wabR-P* operon
2483	CGCAACGAGCGACAACGAACGTTTTAGAGCTAGAAATAG CAAGTTAAAATAAGGC	Primer containing sgRNA to target *pglL*
2484	GTGTGATTCGTGATCGCGTAGTTTTAGAGCTAGAAATAGC AAGTTAAAATAAGGC	Primer containing sgRNA to target *pglL*
2485	AGGTGACCGGTGCTCGACACGTTTTAGAGCTAGAAATAGC AAGTTAAAATAAGGC	Primer containing sgRNA to target *bacA*
2486	GCCCGCGACGATCAGGTGACGTTTTAGAGCTAGAAATAGC AAGTTAAAATAAGGC	Primer containing sgRNA to target *bacA*
2619	ATTAcatatgACCCTCATCGTCACCGG	Used to amplify *hldD* (K562_RS14060)
2620	ATTAtctagaGACCCAGTCTATCGATGCAACG	Used to amplify *hldD* (K562_RS14060)
2627	ATTAcatatgTTCGCCATCATGTTCGG	Used to amplify *waaL* (K562_RS06530)
2628	ATTAtctagaTTCACGATCCGCTCCCC	Used to amplify *waaL* (K562_RS06530)
2629	ATTAcatatgTTGCAATCCAGAGCATCTTGG	Used to amplify *wbiI* (K562_RS15035)
2630	ATTAtctagaCCTGGAGCCACGTCACCC	Used to amplify *wbiI* (K562_RS15035)
2631	ATTAcatatgCGGGATAACATTCAAAGTTCC	Used to amplify *wzm-wzt* (K562_RS15085-80)
2632	ATTAtctagaCGTCGACGCTAAACTCATCATC	Used to amplify *wzm-wzt* (K562_RS15085-80)

2672	AGGCGGCGATGCCGCCAGAGGTTTTAGAGCTAGAAATAG CAAGTTAAAATAAGGC	Primer containing sgRNA to target *wzm-wzt* operon
2673	ATACGGCCAGCAGGAGCAACGTTTTAGAGCTAGAAATAG CAAGTTAAAATAAGGC	Primer containing sgRNA to target *wzm-wzt* operon
2674	AACGGCCACCGGAACACCGCGTTTTAGAGCTAGAAATAGC AAGTTAAAATAAGGC	Primer containing sgRNA to target *waaL*
2675	TCGGGACGAACAACGGCCACGTTTTAGAGCTAGAAATAGC AAGTTAAAATAAGGC	Primer containing sgRNA to target *waaL*
2693	GCTTTGTCGGTTGCGTGA	Colony PCR for *hldD* deletion
2694	CACTGTTGCTCACCCGTTC	Colony PCR for *hldD* deletion
2695	CGCCGATCTTTGGCATCTCTA	Colony PCR for *waaL* deletion
2696	GTCGGTCGGGAAGGCAAC	Colony PCR for *waaL* deletion
2805	GCAGGGTCTCGGTAGGAGTGGTTTTAGAGCTAGAAATAGC AAGTTAAAATAAGGC	Primer containing sgRNA to target *mlaFEDvacJ* operon
2806	GCGAAGTTCCAGCAGGGTCTGTTTTAGAGCTAGAAATAGC AAGTTAAAATAAGGC	Primer containing sgRNA to target *mlaFEDvacJ* operon
2807	TGTTGACGATCGAGATGATGGTTTTAGAGCTAGAAATAGC AAGTTAAAATAAGGC	Primer containing sgRNA to target *mlaCB* operon
2808	GTGCGGCGGAAATCGGTGTAGTTTTAGAGCTAGAAATAGC AAGTTAAAATAAGGC	Primer containing sgRNA to target *mlaCB* operon
2847	TCGTGAAATCCCCGACACGCGTTTTAGAGCTAGAAATAGC AAGTTAAAATAAGGC	Primer containing sgRNA to target K562_RS32470
2848	GCGCTGCATCGCGGTAGCGTGTTTTAGAGCTAGAAATAGC AAGTTAAAATAAGGC	Primer containing sgRNA to target K562_RS32470
3018	CGCGGCGTGGACGGACAGGCGTTTTAGAGCTAGAAATAG CAAGTTAAAATAAGGC	Primer containing sgRNA to targetK562_RS00600
3019	GTTGAGGGCGGGCAGTACCGGTTTTAGAGCTAGAAATAGC AAGTTAAAATAAGGC	Primer containing sgRNA to targetK562_RS00600
3020	GGATTCATACGTGGTGCAGCGTTTTAGAGCTAGAAATAGC AAGTTAAAATAAGGC	Primer containing sgRNA to targetK562_RS07120
3021	ACCGCGACGACGATCACTCGGTTTTAGAGCTAGAAATAGC AAGTTAAAATAAGGC	Primer containing sgRNA to targetK562_RS07120
3022	ATCGGCGTGAGACGCAGGACGTTTTAGAGCTAGAAATAGC AAGTTAAAATAAGGC	Primer containing sgRNA to targetK562_RS07175
3023	AGCGCAATGCCGAAGGCAATGTTTTAGAGCTAGAAATAGC AAGTTAAAATAAGGC	Primer containing sgRNA to targetK562_RS07175
3024	TGCTTCTTGCCGAATTCCCGGTTTTAGAGCTAGAAATAGC AAGTTAAAATAAGGC	Primer containing sgRNA to targetK562_RS09735
3025	ATGCCGCCGAAACGGCGCACGTTTTAGAGCTAGAAATAGC AAGTTAAAATAAGGC	Primer containing sgRNA to targetK562_RS09735

3026	GGCTGGCTCTGGGCGAACGCGTTTTAGAGCTAGAAATAGC AAGTTAAAATAAGGC	Primer containing sgRNA to targetK562_RS09770
3027	TCCGCAATCGATGTGGCGCCGTTTTAGAGCTAGAAATAGC AAGTTAAAATAAGGC	Primer containing sgRNA to targetK562_RS09770
3028	AGGCGTTGTCTGCGCGTAAGGTTTTAGAGCTAGAAATAGC AAGTTAAAATAAGGC	Primer containing sgRNA to targetK562_RS09805
3029	GGTCGCGCTGCCGGCTTGCTGTTTTAGAGCTAGAAATAGC AAGTTAAAATAAGGC	Primer containing sgRNA to targetK562_RS09805
3030	GGCAATCGAGCACAGCGACAGTTTTAGAGCTAGAAATAG CAAGTTAAAATAAGGC	Primer containing sgRNA to targetK562_RS10150
3031	GGCAGGGCAGGAAGCAACGCGTTTTAGAGCTAGAAATAG CAAGTTAAAATAAGGC	Primer containing sgRNA to targetK562_RS10150
3032	GTTCATGGCCGGCGCCGTCTGTTTTAGAGCTAGAAATAGC AAGTTAAAATAAGGC	Primer containing sgRNA to targetK562_RS10200
3033	CAGCGTGCCGTTTTGCGCGCGTTTTAGAGCTAGAAATAGC AAGTTAAAATAAGGC	Primer containing sgRNA to targetK562_RS10200
3034	GGAAGCCATGATCGGAGAGTGTTTTAGAGCTAGAAATAGC AAGTTAAAATAAGGC	Primer containing sgRNA to targetK562_RS11910
3035	AGACGGATGGAAGCCATGATGTTTTAGAGCTAGAAATAGC AAGTTAAAATAAGGC	Primer containing sgRNA to targetK562_RS11910
3036	GTCGGCCGACGCGGACGATGGTTTTAGAGCTAGAAATAGC AAGTTAAAATAAGGC	Primer containing sgRNA to targetK562_RS12040
3037	AGCCGCGAGCCCGTTTCGAGGTTTTAGAGCTAGAAATAGC AAGTTAAAATAAGGC	Primer containing sgRNA to targetK562_RS12040
3038	AGCGGCCGGCCATGCGAAAGGTTTTAGAGCTAGAAATAG CAAGTTAAAATAAGGC	Primer containing sgRNA to targetK562_RS14405
3039	GTCGGTCGAAGCCGCGTGAGGTTTTAGAGCTAGAAATAGC AAGTTAAAATAAGGC	Primer containing sgRNA to targetK562_RS14405
3040	GATCGTGCACGCGGCAAGCAGTTTTAGAGCTAGAAATAGC AAGTTAAAATAAGGC	Primer containing sgRNA to targetK562_RS14515
3041	ATTGCGTTCGGCGACACGATGTTTTAGAGCTAGAAATAGC AAGTTAAAATAAGGC	Primer containing sgRNA to targetK562_RS14515
3042	CCGATATTCTTGAGATCCGTGTTTTAGAGCTAGAAATAGC AAGTTAAAATAAGGC	Primer containing sgRNA to targetK562_RS16355
3043	GAGAATCTTGAGGCGCGGTAGTTTTAGAGCTAGAAATAGC AAGTTAAAATAAGGC	Primer containing sgRNA to targetK562_RS16355
3044	TGTGCGTGGGCCTGCCCGGCGTTTTAGAGCTAGAAATAGC AAGTTAAAATAAGGC	Primer containing sgRNA to targetK562_RS19650
3045	TTCCTGTGCGTGGGCCTGCCGTTTTAGAGCTAGAAATAGC AAGTTAAAATAAGGC	Primer containing sgRNA to targetK562_RS19650
3046	CGCAAACGAAGTGCGGGCGCGTTTTAGAGCTAGAAATAG CAAGTTAAAATAAGGC	Primer containing sgRNA to targetK562_RS19695
3047	CGCCTGCGCAAACGAAGTGCGTTTTAGAGCTAGAAATAGC AAGTTAAAATAAGGC	Primer containing sgRNA to targetK562_RS19695
3048	GACCGACAGCGGTCGCGCCCGTTTTAGAGCTAGAAATAGC AAGTTAAAATAAGGC	Primer containing sgRNA to targetK562_RS20010

3049	AATTCGACCGTCATCGGCAAGTTTTAGAGCTAGAAATAGC AAGTTAAAATAAGGC	Primer containing sgRNA to targetK562_RS20010
3050	CGTCAACAGTCCCCCCAGCGGTTTTAGAGCTAGAAATAGC AAGTTAAAATAAGGC	Primer containing sgRNA to targetK562_RS20035
3051	GCGCCGTCCTCTGCCCACGCGTTTTAGAGCTAGAAATAGC AAGTTAAAATAAGGC	Primer containing sgRNA to targetK562_RS20035
3052	GGGCGTGTCGCCCTGGGCCGGTTTTAGAGCTAGAAATAGC AAGTTAAAATAAGGC	Primer containing sgRNA to targetK562_RS20755
3053	CGGCGCGGGCGTGTCGCCCTGTTTTAGAGCTAGAAATAGC AAGTTAAAATAAGGC	Primer containing sgRNA to targetK562_RS20755
3054	ACGGTGAGTTTCAGCATGCGGTTTTAGAGCTAGAAATAGC AAGTTAAAATAAGGC	Primer containing sgRNA to targetK562_RS21965
3055	AGCGCGCCGACAGCCAGCGCGTTTTAGAGCTAGAAATAGC AAGTTAAAATAAGGC	Primer containing sgRNA to targetK562_RS21965
3056	GCCGGACGCGCCCGGTGCATGTTTTAGAGCTAGAAATAGC AAGTTAAAATAAGGC	Primer containing sgRNA to targetK562_RS25090
3057	AGCGGCGCGGATGCGTTGGCGTTTTAGAGCTAGAAATAGC AAGTTAAAATAAGGC	Primer containing sgRNA to targetK562_RS25090
3058	GGCGAGCGGGGAAAGCAGCGGTTTTAGAGCTAGAAATAG CAAGTTAAAATAAGGC	Primer containing sgRNA to targetK562_RS25215
3059	TTCGGGCAGGTGCCGCGTGTGTTTTAGAGCTAGAAATAGC AAGTTAAAATAAGGC	Primer containing sgRNA to targetK562_RS25215
3060	GAGCTTCAGCTCGTCGGGACGTTTTAGAGCTAGAAATAGC AAGTTAAAATAAGGC	Primer containing sgRNA to targetK562_RS27300
3061	AGATGGCCTTCGGTGCTGGCGTTTTAGAGCTAGAAATAGC AAGTTAAAATAAGGC	Primer containing sgRNA to targetK562_RS27300
3064	CGGACGCCAGCGCCCGAAACGTTTTAGAGCTAGAAATAGC AAGTTAAAATAAGGC	Primer containing sgRNA to targetK562_RS28410
3065	GCATCGGCGAACGCCGCCGCGTTTTAGAGCTAGAAATAGC AAGTTAAAATAAGGC	Primer containing sgRNA to targetK562_RS28410
3066	CCGCATCGCGCCGTACCACCGTTTTAGAGCTAGAAATAGC AAGTTAAAATAAGGC	Primer containing sgRNA to targetK562_RS29155
3067	GAACAGCGCCAACGTGCCAAGTTTTAGAGCTAGAAATAGC AAGTTAAAATAAGGC	Primer containing sgRNA to targetK562_RS29155
3068	GGCACGGCAGGGCGTTCGACGTTTTAGAGCTAGAAATAGC AAGTTAAAATAAGGC	Primer containing sgRNA to targetK562_RS29535
3069	ACGGACGTGTGGTCCGGGCAGTTTTAGAGCTAGAAATAGC AAGTTAAAATAAGGC	Primer containing sgRNA to targetK562_RS29535
3070	CCGAACAGTGCGGCACAGATGTTTTAGAGCTAGAAATAGC AAGTTAAAATAAGGC	Primer containing sgRNA to targetK562_RS30495
3071	GCCGAACGCGCCGAACAGTGGTTTTAGAGCTAGAAATAGC AAGTTAAAATAAGGC	Primer containing sgRNA to targetK562_RS30495
3072	GGCCACGCACATTGCCCCGCGTTTTAGAGCTAGAAATAGC AAGTTAAAATAAGGC	Primer containing sgRNA to targetK562_RS33275
3073	GCCGGCCCTCGCACGTCGATGTTTTAGAGCTAGAAATAGC AAGTTAAAATAAGGC	Primer containing sgRNA to targetK562_RS33275

3074	CGCGTCGGCGCTGCCCATGCGTTTTAGAGCTAGAAATAGC AAGTTAAAATAAGGC	Primer containing sgRNA to targetK562_RS33410
3075	GTCGATTCACGCGTACGAAAGTTTTAGAGCTAGAAATAGC AAGTTAAAATAAGGC	Primer containing sgRNA to targetK562_RS33410

Supplementary Table 10. gBlock fragments used in this work to delete *hldD* and *waaL* (restriction sites are in lowercase)

Name	Sequence (restriction sites in lowercase)	Description
hldD frag	ATTAcccgggATCGTCAAGCCTTCCTGCTGAAGCCGCTCGACGGTGTG<br>CCCGCCCATGCCCTTCACACGCTTGGCGAGATCGTCCGCATTCCTG<br>AACGGACCGCGTGCGCCGCGCTCGTCGAGAATCGCCTTCGCGCGTG<br>CCGGGCCGATGCCCTTGATGCCGACGAGCGCATCCTCGTTCGCGGTG<br>TTGACGTCGACGGCCGCCCAGGCCGACGCTACTGCGCCGAGCATGA<br>CCGCTGCGGCAAACCATTCCCTGAACATGTGCTGGATCTCCGAAGAA<br>AACGGCCGGCGGCTGCCGACCGTGAGACCCAGTCTATCGATGCAAC<br>GCGCCGGTTTAAACCTGGCCGGATAGCCAACGCACGTAACGATCTTG<br>TCGAGATAGTCGTCGATCTCGCAATCGACGAGATTGCGAAACTTGTC<br>TGCGCGGGTCAGGTTGTCGACCGCGATGATGCGCGTCTCGCCGCGC<br>TCGTTGAGCGCCTTGACGATGTTCGCGCCGATAAAACCGGCTGCGCC<br>GGTGACGATGAGGGTCATGATCGTCCTGCCTGAAAAGTGCGAGCCC<br>ACTCGGGCGACGCGCTCGTCGCGCCGCCGAATGCGCTCAGTGAAACA<br>GTTCGTCGTAATCCACCGTGGCCGTGCCGAGCTTGCCGACCACGATG<br>CCTGCCGCGCGATTCGCGAGCACCACCGCGTCGACGAGCGGCACCCC<br>GGCGCCGAGCATCGTCGCGACCGTCGCGATCAtctagaTTA	Enables deletion of the 3' 796bp (of total 993 bp) of *hldD*
waaL frag	ATTATcccgggTGCCGGACGTTACTGGCGCGACGTTCCGTTCGTCGCC<br>GTAACGATCGCGGACATGCCATGCCGCGATCAACCGTATATTTTCC<br>GGCGTCACGTCCAGTGGGTCAGGCGATGCGATCGCGACGCCACGAC<br>ATGCGTCGCGTGGCACGACGGCTCCGCGGGCTGCATGAGCGGGCT<br>GTCGGTGGACGCGGTACGTGGCGCACTGAAACACGTTCTGGTGCAA<br>GTTCGATGACGGCTATTGCGGCGCGTATGGAGCGTCTTCTGTGGATT<br>GGCTGCCCCGTTCTGATGTTCGCCATCATGTTCGGGCACATGGCGGC<br>GTTCGTGAATTGCAGCATGGTGCTGGTCGGGATTGCGCTGATCGAA<br>ACCGGTGCCGGGAATGGCCTACCGCAGCGAGATGCCGCCCGAATTGC<br>TGGCACTCGAGCCGCAGGCGCTGACGCACGCGCACAATCTTTTCCTG<br>AATACGTGGCTACAGACCGGGCGCATCGGCCTGGCAATGCAGGCACT<br>GTTGCTGGCGGCGCTCGTATTCGCGTTCTGGCGACTGCGGAAGGCTG<br>ACCCGTGGATTGCGGCGGGCGGCATCGCGCTCGTGGTCGGGATGGTC<br>ACGAAGAACCTGGTGGACGATTTCATGTGGCAGACGACGATCCTGGC<br>ATTCTGGGCATTTGCCGGGCTGGTGCTCGGGCATGGCGAGCGACGCG<br>CACGCGTGCGTCGCGCGCAACCGGGGAGCGGATCGTGAAtctagaTTAT	Enables deletion of the 5' 785 bp (of total 1230 bp) of *waaL*

Supplementary Table 11. qRT-PCR primers used in this work.

Name	Sequence	Efficiency (%)	Target Gene/Operon
3493	CGG ACC AGC AGA TCC AGT AC	104.0	*mlaCB*
3494	GGT AAT CGA TCT GGA CCG GC		*mlaCB*
3497	ATC CTG CGT TGG TGG ATC TG	101.7	*wbiFGHI*
3498	CGT CCG TTT CGT TGA TGC AG		*wbiFGHI*
3501	GGT GAC GCG ACA ATG TTC AG	101.5	*vioAwbxFED*
3502	AAT CGA CGC GCT TCT TCA GT		*vioAwbxFED*
3505	GTG GGC AAC TAT CGG GCA A	105.1	*ogcABEI*
3506	CGC CCT CGT TAA GCT CGT AA		*ogcABEI*
3509	CTG TTC GGC GAA TAC AAC GG	104.2	*hldD*
3510	TTC ACC TTG GTC ACG TCC TC		*hldD*
3513	CCG TCA CAA ATG GCT GGG TA	103.0	*waaL-wabQ*
3514	GCT CGA GTG CCA GCA ATT C		*waaL-wabQ*
3521	CAC GAC CTG ACC GAC GAA G	97.3	*ispDF*
3522	CGT GAC CTT GAA GTT GCG C		*ispDF*
3525	GTT TGA CGC TCA CCA TCG C	94.9	*uppS-rseP*
3526	ATG CAT CCT CGG TCA CTT CG		*uppS-rseP*
3529	GGC TGC GGA CCA ACA ACA AT	98.1	*penR*
3530	CAG TTG CCT TCG CCG AAA C		*penR*
3537	GCA CCG CAA GTA TTC GAA CG	102.9	*ampC*
3538	CGT ATG CGT CAT CCC GAT CG		*ampC*
3543	TCC GCA CAT CCA GAT CGA AC	108.6	K562_RS32470
3544	GAT CAG CAG CCC TTC CGA G		K562_RS32470
3619	CGCGTTGTTTACCGACATGT	104.9	*mlaFEDvacJ*
3620	TCTTCGGGCAGATCGGTATG		*mlaFEDvacJ*
3621	CGGTACCCAGATCGTGCAG	103.6	*pglL*
3622	ACCGCGATGTTGTACACCAC		*pglL*
1238	GCG TCA GAT CTT GCA CAT CG	97.9	*rpoD*
1239	GTG ATC TTC TCG GCC CAG TC		*rpoD*

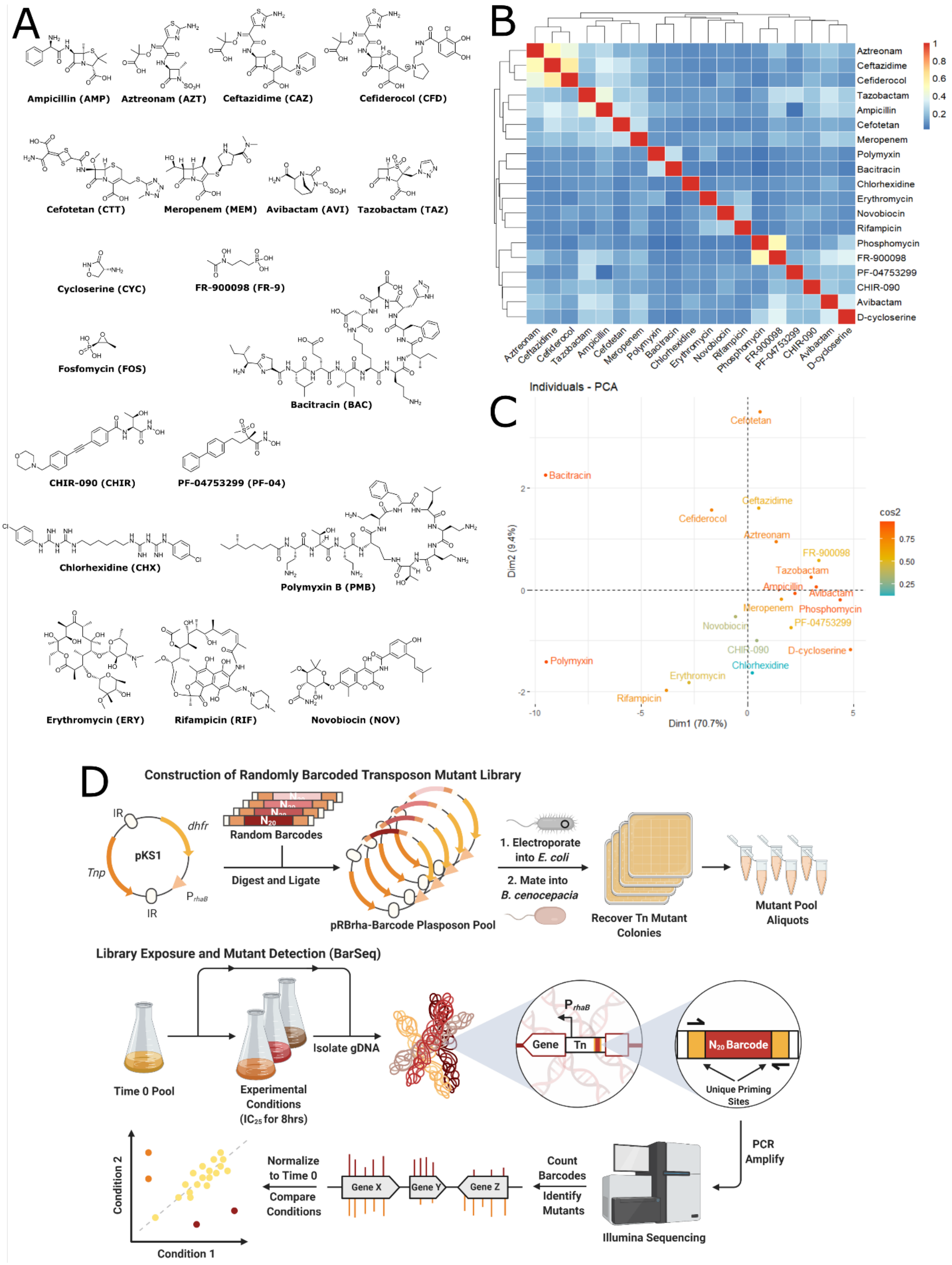
A
Ampicillin (AMP)
Aztreonam (AZT)
Ceftazidime (CAZ)
Cefiderocol (CFD)
Cefotetan (CTT)
Meropenem (MEM)
Avibactam (AVI)
Tazobactam (TAZ)
Cycloserine (CYC)
FR-900098 (FR-9)
Fosfomycin (FOS)
Bacitracin (BAC)
CHIR-090 (CHIR)
PF-04753299 (PF-04)
Chlorhexidine (CHX)
Polymyxin B (PMB)
Erythromycin (ERY)
Rifampicin (RIF)
Novobiocin (NOV)
B
Aztreonam
Ceftazidime
Cefiderocol
Tazobactam
Ampicillin
Cefotetan
Meropenem
Polymyxin
Bacitracin
Chlorhexidine
Erythromycin
Novobiocin
Rifampicin
Phosphomycin
FR-900098
PF-04753299
CHIR-090
Avibactam
D-cycloserine
1
0.8
0.6
0.4
0.2
C
Individuals - PCA
Cefotetan
Bacitracin
Ceftazidime
Cefiderocol
Aztreonam
FR-900098
Tazobactam
Ampicillin
Avibactam
Meropenem
Phosphomycin
Novobiocin
PF-04753299
CHIR-090
D-cycloserine
Polymyxin
Chlorhexidine
Erythromycin
Rifampicin
Dim2 (9.4%)
Dim1 (70.7%)
cos2
0.75
0.50
0.25
D
Construction of Randomly Barcoded Transposon Mutant Library
IR
dhfr
pKS1
Tnp
IR
Random Barcodes
Digest and Ligate
pRBrha-Barcode Plasposon Pool
1. Electroporate into E. coli
2. Mate into B. cenocepacia
Recover Tn Mutant Colonies
Mutant Pool Aliquots
Library Exposure and Mutant Detection (BarSeq)
Time 0 Pool
Experimental Conditions (IC25 for 8hrs)
Isolate gDNA
Gene
Tn
N20 Barcode
Unique Priming Sites
PCR Amplify
Illumina Sequencing
Count Barcodes
Identify Mutants
Gene X
Gene Y
Gene Z
Normalize to Time 0
Compare Conditions
Condition 2
Condition 1

Supplementary Figure 1. Schematic of creating randomly-barcoded transposon mutants and exposure to an antibiotic panel. A) Structure of the antibiotics used in the BarSeq experiment. B) 2-D hierarchical clustering of atom pair Tanimoto similarity scoring from the ChemMine Tools package (Backman et al., 2011). Warmer colours indicate a higher degree of similarity. C) Principal component analysis based on chemical properties from the ChemMine Tools package. Warmer colours indicate better representation by the principal components. D) DNA fragments containing random 20 bp barcodes flanked by priming and restriction sites are ligated into the parent plasposon pKS1. The resultant plasposon pool is electroporated into E. coli and then introduced into K56-2 by triparental mating. Mutant colonies are recovered and stored until needed. After growth in a desired condition, genomic DNA is isolated and used as template for one-step PCR to amplify barcodes and add flow cell adapters and sequencing indices. Prepared DNA libraries are then sequenced on an Illumina platform (BarSeq). Barcode abundance is counted and compared between conditions by a bioinformatic pipeline. Image created with BioRendr.

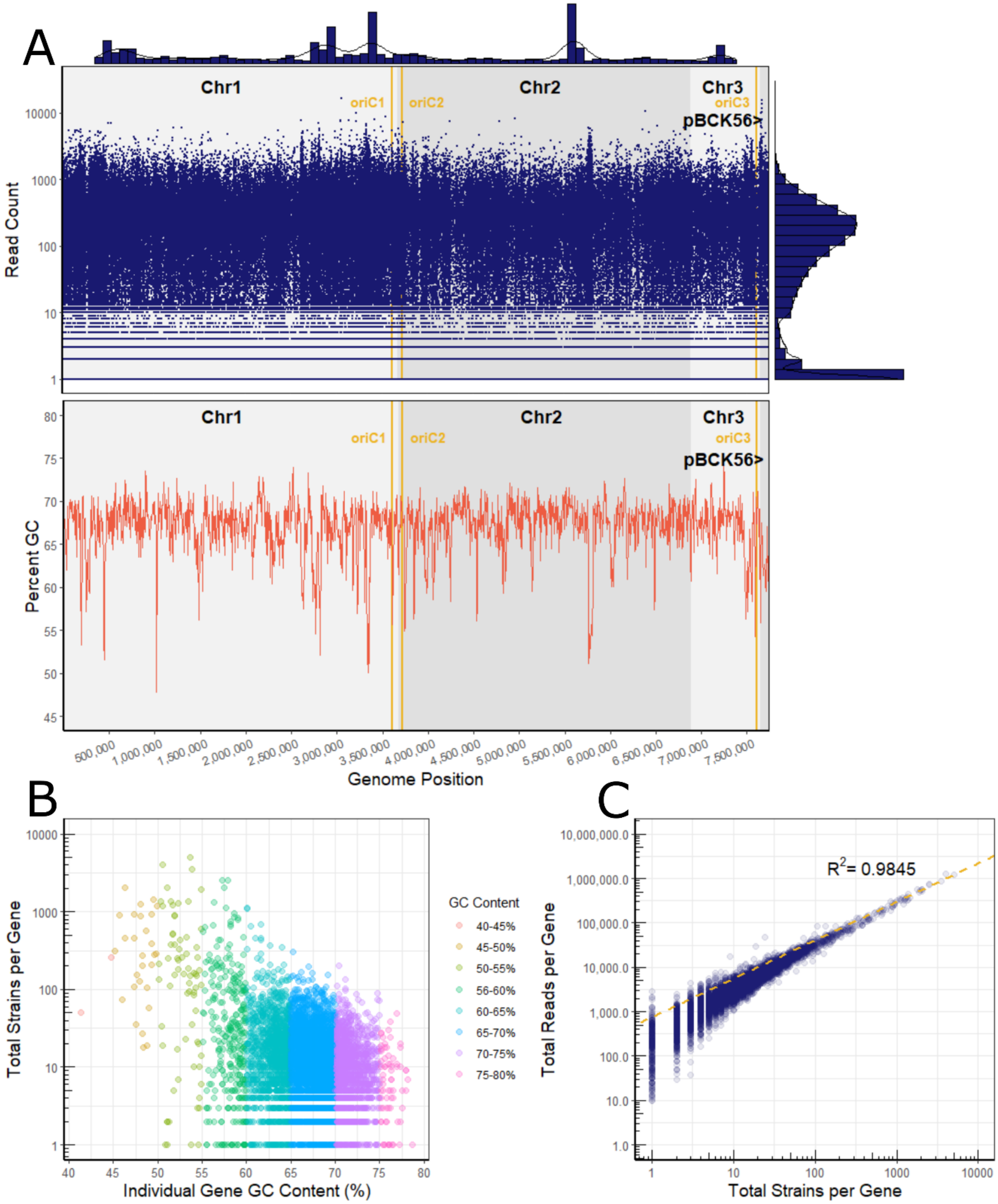
A
Chr1
Chr2
Chr3
oriC1
oriC2
oriC3
pBCK56>
Read Count
Percent GC
Genome Position
B
Total Strains per Gene
Individual Gene GC Content (%)
GC Content
40-45%
45-50%
50-55%
56-60%
60-65%
65-70%
70-75%
75-80%
C
R^2= 0.9845
Total Reads per Gene
Total Strains per Gene

Supplementary Figure 2. Transposon insertion density across the K56-2 genome. A). Each insertion in the library was mapped along with the Illumina read count at that site. The marginal histograms represent 1-D point density in the x and y dimensions of the plot. The x-marginal histogram has a bin width of 100,000 bp and shows insertion density. The y-marginal histogram splits each log_{10} into 5 bins and shows read count density. The chromosomal origins (OriC) were identified by homology from *B. cenocepacia* J2315 using DoriC 10.0 [14]. The GC_content perl script (https://github.com/DamienFr/GC_content_in_sliding_window) was used to calculate GC content with a 5,000 bp window and 5,000 bp step size. B) Gene-level breakdown of the number of strains per gene versus GC content. Each point is coloured by binned GC-content of the gene. C) The number of reads per gene versus the number of strains the gene is represented by in the library. The dashed yellow line is a linear regression showing the relationship between the number of reads and number of strains for each gene.

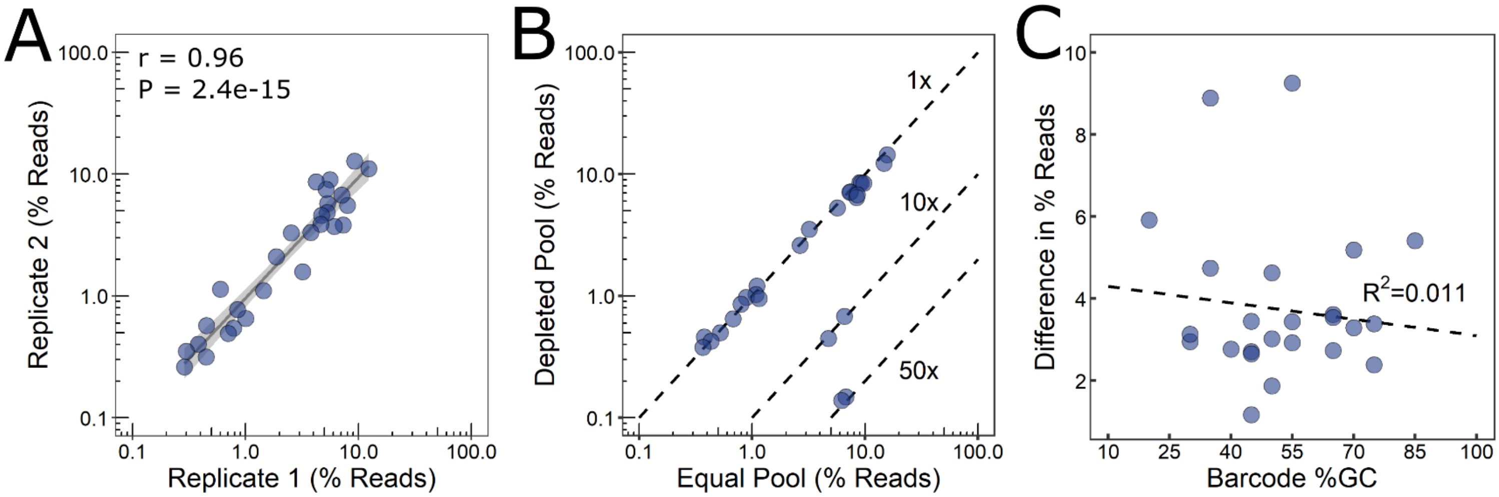

Supplementary Figure 3. Validation of BarSeq to reproducibly and accurately determine barcoded transposon mutant abundance. A) Reproducibility of BarSeq shown between two replicates of artificial equal pools of 30 randomly selected mutants. Each point represents the value from a single replicate. The grey line shows a linear regression with 95% confidence interval in lighter grey. The r and P-values are from Pearson's correlation test (two-sided). B) Detection of changes in barcode abundance in artificial pools of 30 mutants (equal vs depleted at 10x and 50x). Each point is an average of two replicates. Dashed lines indicate expected mutant abundance in the two pools: 1x, 10x, or 50x. C) Absolute difference between the observed and expected barcode abundance (in percent of reads) for the equal pool replicates as a function of individual barcode GC-content. If truly equal, all barcodes would be present at ~3%. The dashed line shows a linear regression.

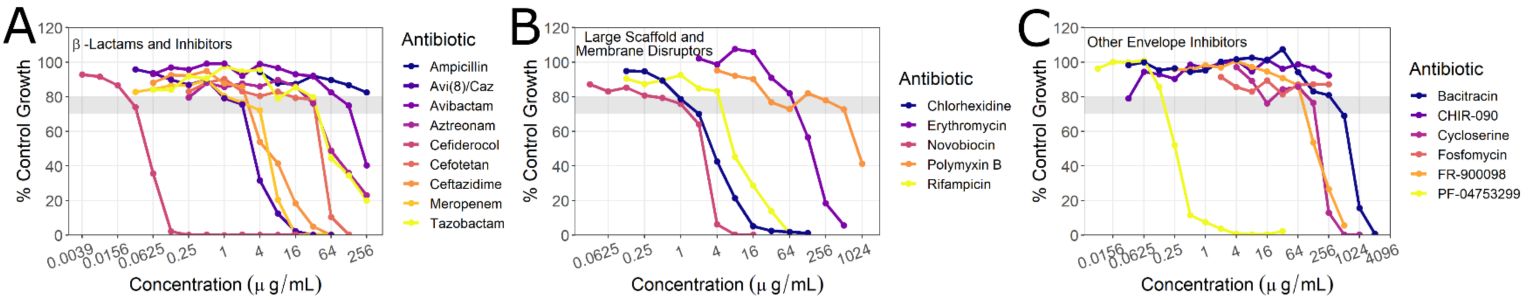

Supplementary Figure 4. Growth dose-response curves of wild-type K56-2 to antibiotics in the panel. Antibiotics are grouped by A) β-lactams and β-lactamase inhibitors, B) large scaffold antibiotics and membrane disruptors, and C) other envelope inhibitors. The grey box indicates the target region of 20 – 30% growth inhibition relative to the no-antibiotic control. Points are averages of three biological replicates. Cells were grown in LB to more closely represent conditions of the mutant library exposure.

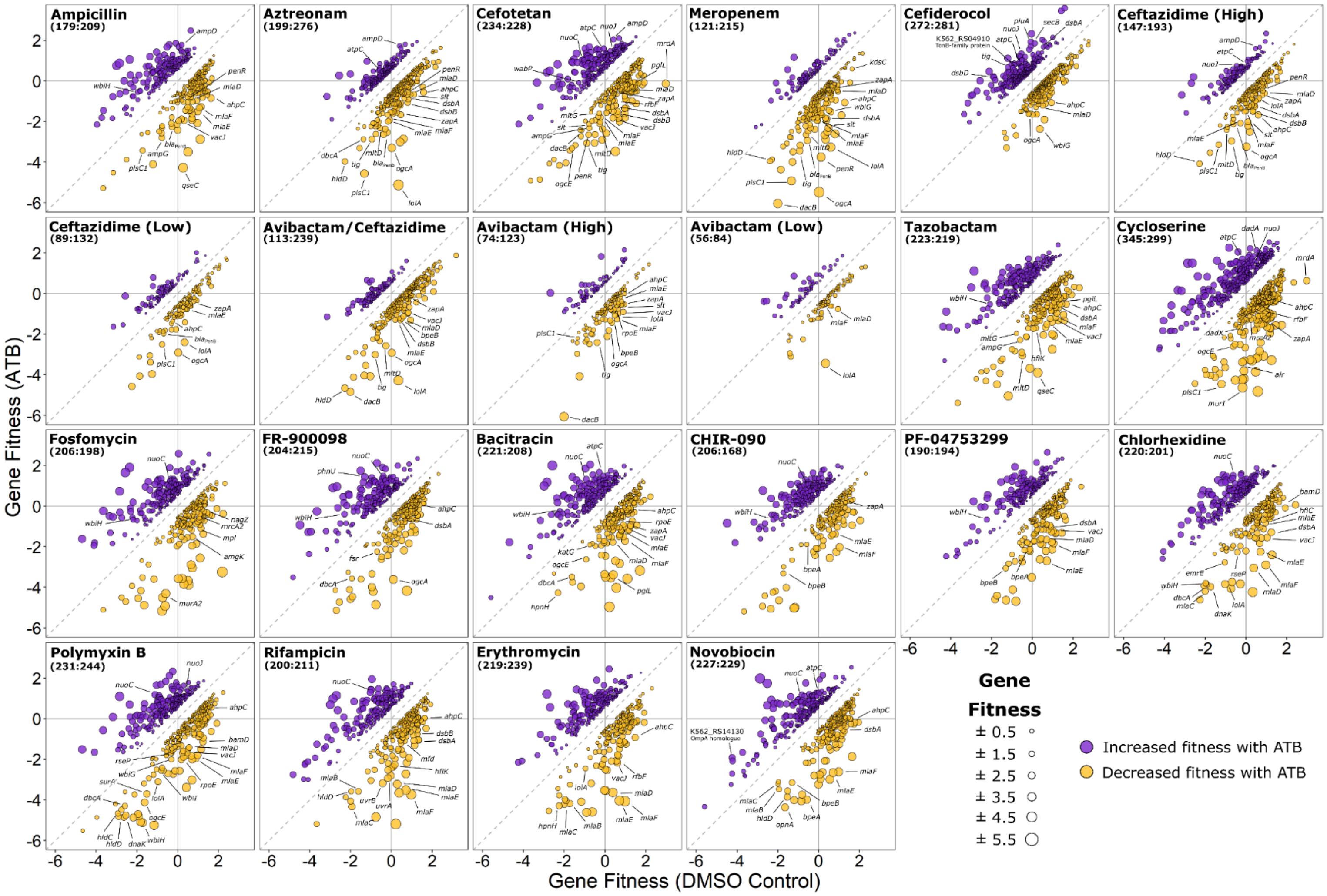

Supplementary Figure 5. Gene fitness profiles of K56-2 transposon mutant library exposed to cell envelope-targeting antibiotic panel. Each point represents a gene that when disrupted by a transposon, affected fitness of the mutant in the presence of an antibiotic versus the DMSO control (two-sided t-test; $P < 0.05$; only genes with fitness effects greater than 0.5 or less than -0.5 were considered). The points are coloured based on positive (increased fitness; purple) or negative (decreased fitness; gold) interactions between the genes and tested antibiotics. The absolute difference in fitness score for a gene in each condition versus the DMSO control is given by the size of the point. Select genes of interest are indicated and named. The total number of genes in each condition with altered fitness (increased:decreased) is shown.

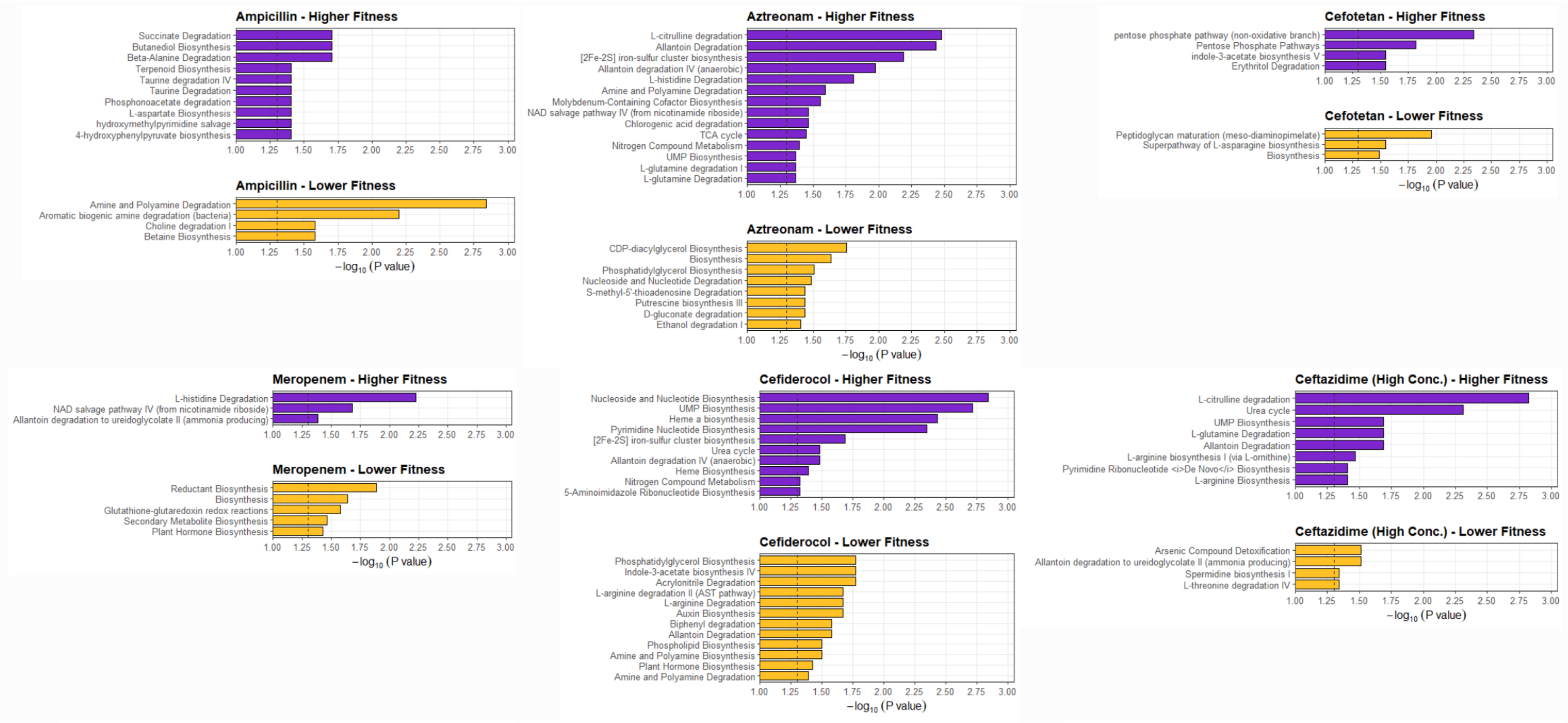

Ampicillin - Higher Fitness
Succinate Degradation
Butanediol Biosynthesis
Beta-Alanine Degradation
Terpenoid Biosynthesis
Taurine degradation IV
Taurine Degradation
Phosphonoacetate degradation
L-aspartate Biosynthesis
hydroxymethylpyrimidine salvage
4-hydroxyphenylpyruvate biosynthesis
1.00 1.25 1.50 1.75 2.00 2.25 2.50 2.75 3.00
Ampicillin - Lower Fitness
Amine and Polyamine Degradation
Aromatic biogenic amine degradation (bacteria)
Choline degradation I
Betaine Biosynthesis
1.00 1.25 1.50 1.75 2.00 2.25 2.50 2.75 3.00
$-\log_{10}$ (P value)
Aztreonam - Higher Fitness
L-citrulline degradation
Allantoin Degradation
[2Fe-2S] iron-sulfur cluster biosynthesis
Allantoin degradation IV (anaerobic)
L-histidine Degradation
Amine and Polyamine Degradation
Molybdenum-Containing Cofactor Biosynthesis
NAD salvage pathway IV (from nicotinamide riboside)
Chlorogenic acid degradation
TCA cycle
Nitrogen Compound Metabolism
UMP Biosynthesis
L-glutamine degradation I
L-glutamine Degradation
1.00 1.25 1.50 1.75 2.00 2.25 2.50 2.75 3.00
Aztreonam - Lower Fitness
CDP-diacylglycerol Biosynthesis
Biosynthesis
Phosphatidylglycerol Biosynthesis
Nucleoside and Nucleotide Degradation
S-methyl-5'-thioadenosine Degradation
Putrescine biosynthesis III
D-gluconate degradation
Ethanol degradation I
1.00 1.25 1.50 1.75 2.00 2.25 2.50 2.75 3.00
$-\log_{10}$ (P value)
Cefotetan - Higher Fitness
pentose phosphate pathway (non-oxidative branch)
Pentose Phosphate Pathways
indole-3-acetate biosynthesis V
Erythritol Degradation
1.00 1.25 1.50 1.75 2.00 2.25 2.50 2.75 3.00
Cefotetan - Lower Fitness
Peptidoglycan maturation (meso-diaminopimelate)
Superpathway of L-asparagine biosynthesis
Biosynthesis
1.00 1.25 1.50 1.75 2.00 2.25 2.50 2.75 3.00
$-\log_{10}$ (P value)
Meropenem - Higher Fitness
L-histidine Degradation
NAD salvage pathway IV (from nicotinamide riboside)
Allantoin degradation to ureidoglycolate II (ammonia producing)
1.00 1.25 1.50 1.75 2.00 2.25 2.50 2.75 3.00
Meropenem - Lower Fitness
Reductant Biosynthesis
Biosynthesis
Glutathione-glutaredoxin redox reactions
Secondary Metabolite Biosynthesis
Plant Hormone Biosynthesis
1.00 1.25 1.50 1.75 2.00 2.25 2.50 2.75 3.00
$-\log_{10}$ (P value)
Cefiderocol - Higher Fitness
Nucleoside and Nucleotide Biosynthesis
UMP Biosynthesis
Heme a biosynthesis
Pyrimidine Nucleotide Biosynthesis
[2Fe-2S] iron-sulfur cluster biosynthesis
Urea cycle
Allantoin degradation IV (anaerobic)
Heme Biosynthesis
Nitrogen Compound Metabolism
5-Aminoimidazole Ribonucleotide Biosynthesis
1.00 1.25 1.50 1.75 2.00 2.25 2.50 2.75 3.00
Cefiderocol - Lower Fitness
Phosphatidylglycerol Biosynthesis
Indole-3-acetate biosynthesis IV
Acrylonitrile Degradation
L-arginine degradation II (AST pathway)
L-arginine Degradation
Auxin Biosynthesis
Biphenyl degradation
Allantoin Degradation
Phospholipid Biosynthesis
Amine and Polyamine Biosynthesis
Plant Hormone Biosynthesis
Amine and Polyamine Degradation
1.00 1.25 1.50 1.75 2.00 2.25 2.50 2.75 3.00
$-\log_{10}$ (P value)
Ceftazidime (High Conc.) - Higher Fitness
L-citrulline degradation
Urea cycle
UMP Biosynthesis
L-glutamine Degradation
Allantoin Degradation
L-arginine biosynthesis I (via L-ornithine)
Pyrimidine Ribonucleotide <i>De Novo</i> Biosynthesis
L-arginine Biosynthesis
1.00 1.25 1.50 1.75 2.00 2.25 2.50 2.75 3.00
Ceftazidime (High Conc.) - Lower Fitness
Arsenic Compound Detoxification
Allantoin degradation to ureidoglycolate II (ammonia producing)
Spermidine biosynthesis I
L-threonine degradation IV
1.00 1.25 1.50 1.75 2.00 2.25 2.50 2.75 3.00
$-\log_{10}$ (P value)

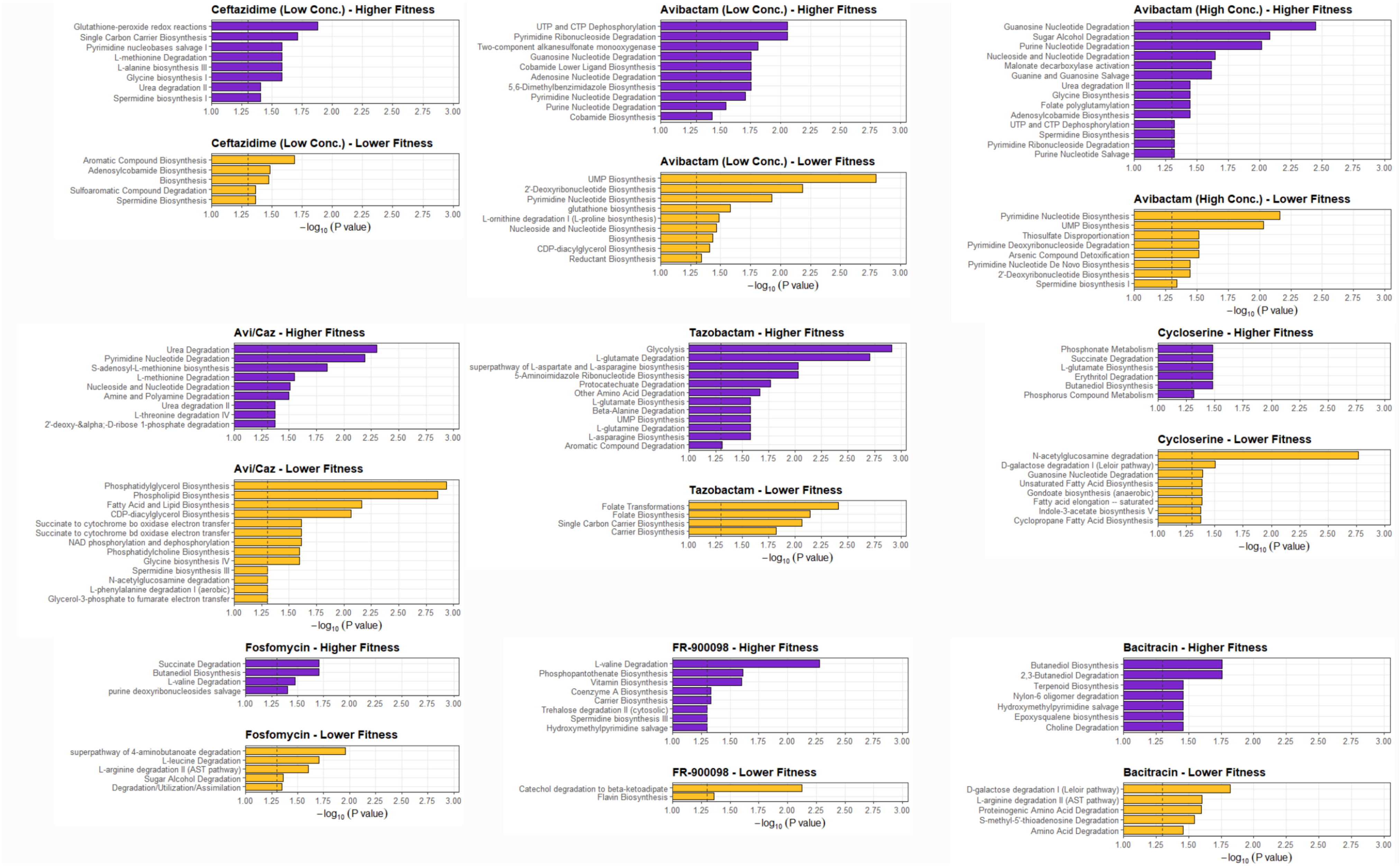

Ceftazidime (Low Conc.) - Higher Fitness
Glutathione-peroxide redox reactions
Single Carbon Carrier Biosynthesis
Pyrimidine nucleobases salvage I
L-methionine Degradation
L-alanine biosynthesis III
Glycine biosynthesis I
Urea degradation II
Spermidine biosynthesis I
Ceftazidime (Low Conc.) - Lower Fitness
Aromatic Compound Biosynthesis
Adenosylcobamide Biosynthesis
Biosynthesis
Sulfoaromatic Compound Degradation
Spermidine Biosynthesis
Avibactam (Low Conc.) - Higher Fitness
UTP and CTP Dephosphorylation
Pyrimidine Ribonucleoside Degradation
Two-component alkanesulfonate monooxygenase
Guanosine Nucleotide Degradation
Cobamide Lower Ligand Biosynthesis
Adenosine Nucleotide Degradation
5,6-Dimethylbenzimidazole Biosynthesis
Pyrimidine Nucleotide Degradation
Purine Nucleotide Degradation
Cobamide Biosynthesis
Avibactam (Low Conc.) - Lower Fitness
UMP Biosynthesis
2'-Deoxyribonucleotide Biosynthesis
Pyrimidine Nucleotide Biosynthesis
glutathione biosynthesis
L-ornithine degradation I (L-proline biosynthesis)
Nucleoside and Nucleotide Biosynthesis
Biosynthesis
CDP-diacylglycerol Biosynthesis
Reductant Biosynthesis
Avibactam (High Conc.) - Higher Fitness
Guanosine Nucleotide Degradation
Sugar Alcohol Degradation
Purine Nucleotide Degradation
Nucleoside and Nucleotide Degradation
Malonate decarboxylase activation
Guanine and Guanosine Salvage
Urea degradation II
Glycine Biosynthesis
Folate polyglutamylation
Adenosylcobamide Biosynthesis
UTP and CTP Dephosphorylation
Spermidine Biosynthesis
Pyrimidine Ribonucleoside Degradation
Purine Nucleotide Salvage
Avibactam (High Conc.) - Lower Fitness
Pyrimidine Nucleotide Biosynthesis
UMP Biosynthesis
Thiosulfate Disproportionation
Pyrimidine Deoxyribonucleoside Degradation
Arsenic Compound Detoxification
Pyrimidine Nucleotide De Novo Biosynthesis
2'-Deoxyribonucleotide Biosynthesis
Spermidine biosynthesis I
Avi/Caz - Higher Fitness
Urea Degradation
Pyrimidine Nucleotide Degradation
S-adenosyl-L-methionine biosynthesis
L-methionine Degradation
Nucleoside and Nucleotide Degradation
Amine and Polyamine Degradation
Urea degradation II
L-threonine degradation IV
2'-deoxy-α-D-ribose 1-phosphate degradation
Avi/Caz - Lower Fitness
Phosphatidylglycerol Biosynthesis
Phospholipid Biosynthesis
Fatty Acid and Lipid Biosynthesis
CDP-diacylglycerol Biosynthesis
Succinate to cytochrome bo oxidase electron transfer
Succinate to cytochrome bd oxidase electron transfer
NAD phosphorylation and dephosphorylation
Phosphatidylcholine Biosynthesis
Glycine biosynthesis IV
Spermidine biosynthesis III
N-acetylglucosamine degradation
L-phenylalanine degradation I (aerobic)
Glycerol-3-phosphate to fumarate electron transfer
Tazobactam - Higher Fitness
Glycolysis
L-glutamate Degradation
superpathway of L-aspartate and L-asparagine biosynthesis
5-Aminoimidazole Ribonucleotide Biosynthesis
Protocatechuate Degradation
Other Amino Acid Degradation
L-glutamate Biosynthesis
Beta-Alanine Degradation
UMP Biosynthesis
L-glutamine Degradation
L-asparagine Biosynthesis
Aromatic Compound Degradation
Tazobactam - Lower Fitness
Folate Transformations
Folate Biosynthesis
Single Carbon Carrier Biosynthesis
Carrier Biosynthesis
Cycloserine - Higher Fitness
Phosphonate Metabolism
Succinate Degradation
L-glutamate Biosynthesis
Erythritol Degradation
Butanediol Biosynthesis
Phosphorus Compound Metabolism
Cycloserine - Lower Fitness
N-acetylglucosamine degradation
D-galactose degradation I (Leloir pathway)
Guanosine Nucleotide Degradation
Unsaturated Fatty Acid Biosynthesis
Gondoate biosynthesis (anaerobic)
Fatty acid elongation -- saturated
Indole-3-acetate biosynthesis V
Cyclopropane Fatty Acid Biosynthesis
Fosfomycin - Higher Fitness
Succinate Degradation
Butanediol Biosynthesis
L-valine Degradation
purine deoxyribonucleosides salvage
Fosfomycin - Lower Fitness
superpathway of 4-aminobutanoate degradation
L-leucine Degradation
L-arginine degradation II (AST pathway)
Sugar Alcohol Degradation
Degradation/Utilization/Assimilation
FR-900098 - Higher Fitness
L-valine Degradation
Phosphopantothenate Biosynthesis
Vitamin Biosynthesis
Coenzyme A Biosynthesis
Carrier Biosynthesis
Trehalose degradation II (cytosolic)
Spermidine biosynthesis III
Hydroxymethylpyrimidine salvage
FR-900098 - Lower Fitness
Catechol degradation to beta-ketoadipate
Flavin Biosynthesis
Bacitracin - Higher Fitness
Butanediol Biosynthesis
2,3-Butanediol Degradation
Terpenoid Biosynthesis
Nylon-6 oligomer degradation
Hydroxymethylpyrimidine salvage
Epoxysqualene biosynthesis
Choline Degradation
Bacitracin - Lower Fitness
D-galactose degradation I (Leloir pathway)
L-arginine degradation II (AST pathway)
Proteinogenic Amino Acid Degradation
S-methyl-5'-thioadenosine Degradation
Amino Acid Degradation
1.00 1.25 1.50 1.75 2.00 2.25 2.50 2.75 3.00
$-\log_{10}$ (P value)

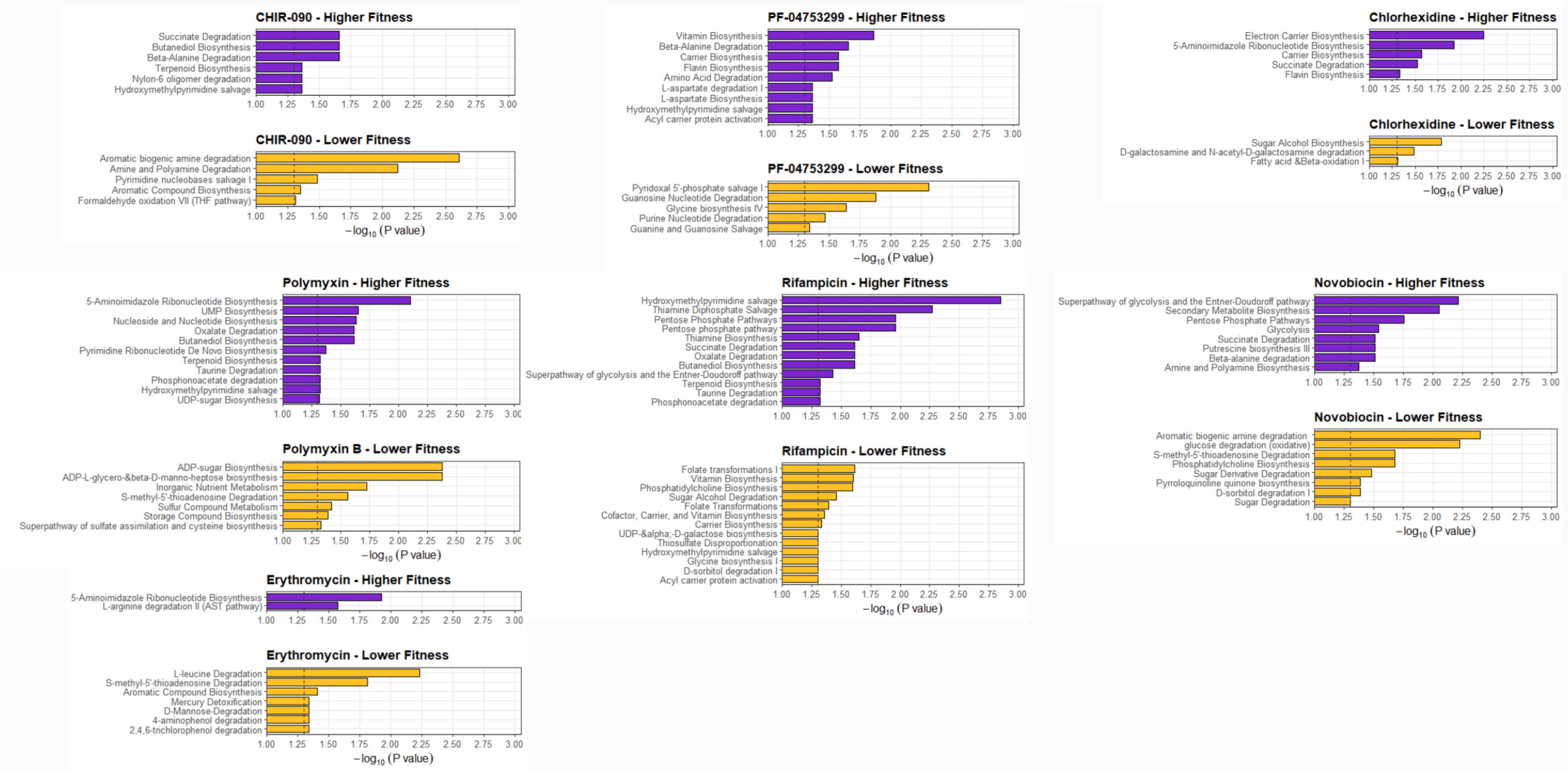

Supplementary Figure 6. BioCyc pathway enrichment of genes affecting fitness in the presence of the antibiotic panel. Genes with fitness scores either above 0.5 or below -0.5 were submitted to BioCyc for enrichment analysis (Fisher exact two-sided test without multiple testing correction) (against K56-2 genome from GenBank accession GCA_000333155.2). Enrichments from genes with positive and negative fitness scores are given in purple and gold, respectively. The dashed line indicates P = 0.05.

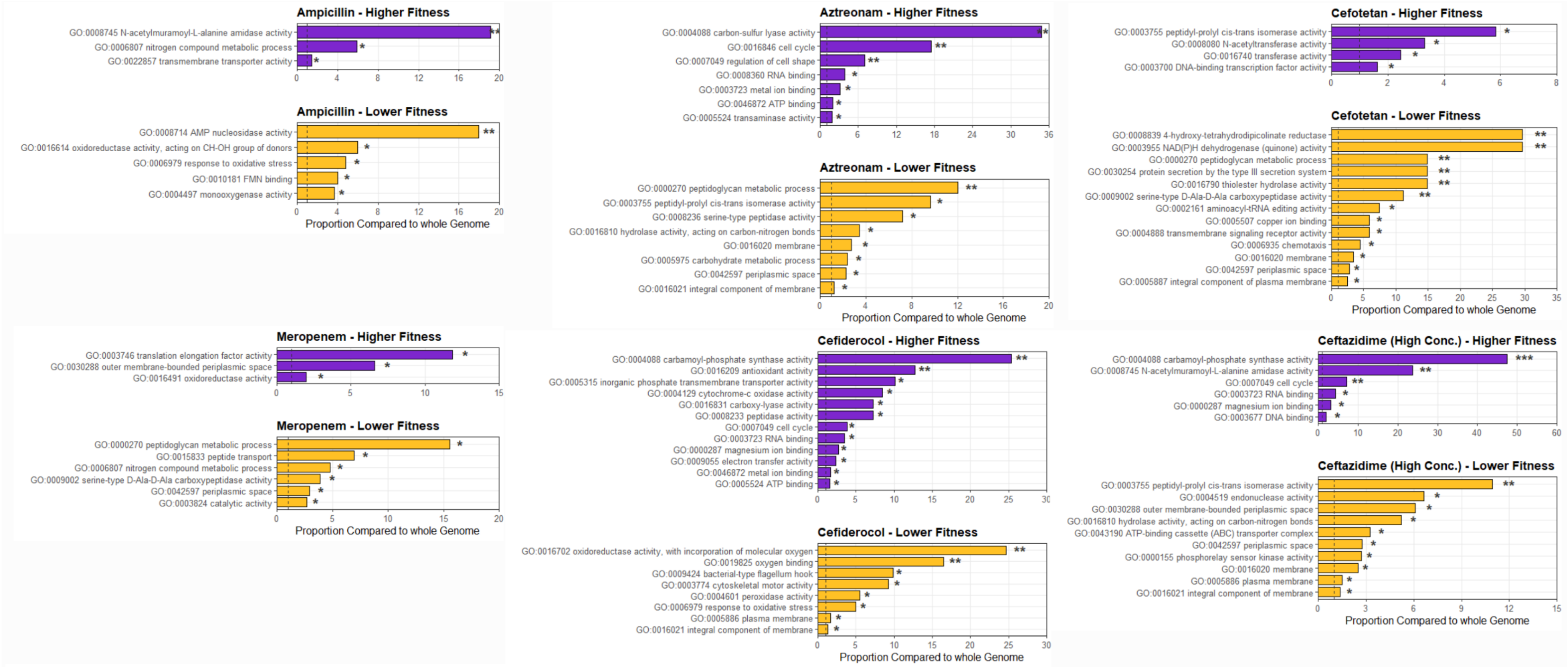

Ampicillin - Higher Fitness
GO:0008745 N-acetylmuramoyl-L-alanine amidase activity
GO:0006807 nitrogen compound metabolic process
GO:0022857 transmembrane transporter activity
Ampicillin - Lower Fitness
GO:0008714 AMP nucleosidase activity
GO:0016614 oxidoreductase activity, acting on CH-OH group of donors
GO:0006979 response to oxidative stress
GO:0010181 FMN binding
GO:0004497 monooxygenase activity
Proportion Compared to whole Genome
Aztreonam - Higher Fitness
GO:0004088 carbon-sulfur lyase activity
GO:0016846 cell cycle
GO:0007049 regulation of cell shape
GO:0008360 RNA binding
GO:0003723 metal ion binding
GO:0046872 ATP binding
GO:0005524 transaminase activity
Aztreonam - Lower Fitness
GO:0000270 peptidoglycan metabolic process
GO:0003755 peptidyl-prolyl cis-trans isomerase activity
GO:0008236 serine-type peptidase activity
GO:0016810 hydrolase activity, acting on carbon-nitrogen bonds
GO:0016020 membrane
GO:0005975 carbohydrate metabolic process
GO:0042597 periplasmic space
GO:0016021 integral component of membrane
Proportion Compared to whole Genome
Cefotetan - Higher Fitness
GO:0003755 peptidyl-prolyl cis-trans isomerase activity
GO:0008080 N-acetyltransferase activity
GO:0016740 transferase activity
GO:0003700 DNA-binding transcription factor activity
Cefotetan - Lower Fitness
GO:0008839 4-hydroxy-tetrahydrodipicolinate reductase
GO:0003955 NAD(P)H dehydrogenase (quinone) activity
GO:0000270 peptidoglycan metabolic process
GO:0030254 protein secretion by the type III secretion system
GO:0016790 thiolester hydrolase activity
GO:0009002 serine-type D-Ala-D-Ala carboxypeptidase activity
GO:0002161 aminoacyl-tRNA editing activity
GO:0005507 copper ion binding
GO:0004888 transmembrane signaling receptor activity
GO:0006935 chemotaxis
GO:0016020 membrane
GO:0042597 periplasmic space
GO:0005887 integral component of plasma membrane
Proportion Compared to whole Genome
Meropenem - Higher Fitness
GO:0003746 translation elongation factor activity
GO:0030288 outer membrane-bounded periplasmic space
GO:0016491 oxidoreductase activity
Meropenem - Lower Fitness
GO:0000270 peptidoglycan metabolic process
GO:0015833 peptide transport
GO:0006807 nitrogen compound metabolic process
GO:0009002 serine-type D-Ala-D-Ala carboxypeptidase activity
GO:0042597 periplasmic space
GO:0003824 catalytic activity
Proportion Compared to whole Genome
Cefiderocol - Higher Fitness
GO:0004088 carbamoyl-phosphate synthase activity
GO:0016209 antioxidant activity
GO:0005315 inorganic phosphate transmembrane transporter activity
GO:0004129 cytochrome-c oxidase activity
GO:0016831 carboxy-lyase activity
GO:0008233 peptidase activity
GO:0007049 cell cycle
GO:0003723 RNA binding
GO:0000287 magnesium ion binding
GO:0009055 electron transfer activity
GO:0046872 metal ion binding
GO:0005524 ATP binding
Cefiderocol - Lower Fitness
GO:0016702 oxidoreductase activity, with incorporation of molecular oxygen
GO:0019825 oxygen binding
GO:0009424 bacterial-type flagellum hook
GO:0003774 cytoskeletal motor activity
GO:0004601 peroxidase activity
GO:0006979 response to oxidative stress
GO:0005886 plasma membrane
GO:0016021 integral component of membrane
Proportion Compared to whole Genome
Ceftazidime (High Conc.) - Higher Fitness
GO:0004088 carbamoyl-phosphate synthase activity
GO:0008745 N-acetylmuramoyl-L-alanine amidase activity
GO:0007049 cell cycle
GO:0003723 RNA binding
GO:0000287 magnesium ion binding
GO:0003677 DNA binding
Ceftazidime (High Conc.) - Lower Fitness
GO:0003755 peptidyl-prolyl cis-trans isomerase activity
GO:0004519 endonuclease activity
GO:0030288 outer membrane-bounded periplasmic space
GO:0016810 hydrolase activity, acting on carbon-nitrogen bonds
GO:0043190 ATP-binding cassette (ABC) transporter complex
GO:0042597 periplasmic space
GO:0000155 phosphorelay sensor kinase activity
GO:0016020 membrane
GO:0005886 plasma membrane
GO:0016021 integral component of membrane
Proportion Compared to whole Genome

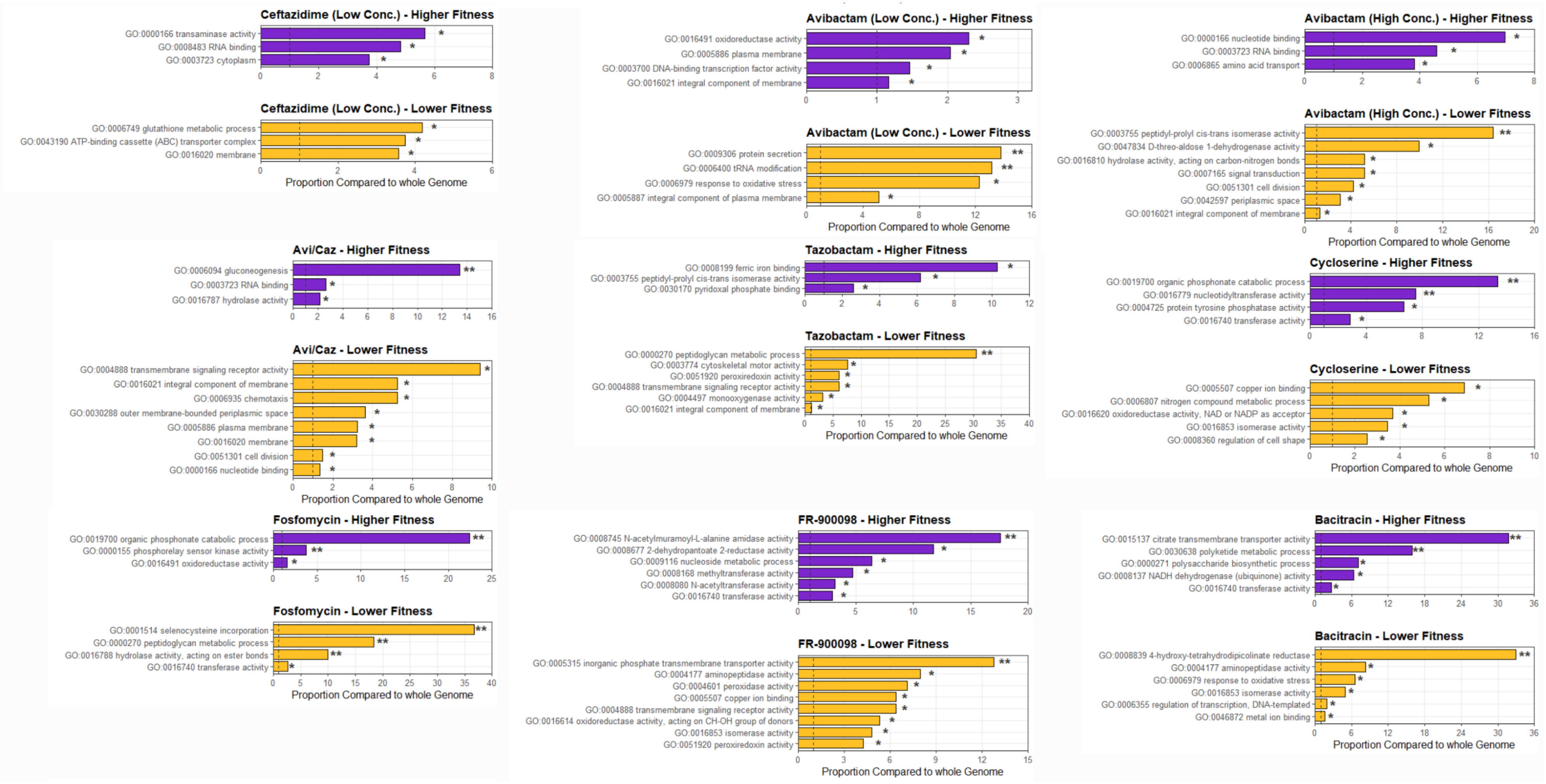
Ceftazidime (Low Conc.) - Higher Fitness
GO:0000166 transaminase activity
GO:0008483 RNA binding
GO:0003723 cytoplasm
Ceftazidime (Low Conc.) - Lower Fitness
GO:0006749 glutathione metabolic process
GO:0043190 ATP-binding cassette (ABC) transporter complex
GO:0016020 membrane
Proportion Compared to whole Genome
Avibactam (Low Conc.) - Higher Fitness
GO:0016491 oxidoreductase activity
GO:0005886 plasma membrane
GO:0003700 DNA-binding transcription factor activity
GO:0016021 integral component of membrane
Avibactam (Low Conc.) - Lower Fitness
GO:0009306 protein secretion
GO:0006400 tRNA modification
GO:0006979 response to oxidative stress
GO:0005887 integral component of plasma membrane
Proportion Compared to whole Genome
Avibactam (High Conc.) - Higher Fitness
GO:0000166 nucleotide binding
GO:0003723 RNA binding
GO:0006865 amino acid transport
Avibactam (High Conc.) - Lower Fitness
GO:0003755 peptidyl-prolyl cis-trans isomerase activity
GO:0047834 D-threo-aldose 1-dehydrogenase activity
GO:0016810 hydrolase activity, acting on carbon-nitrogen bonds
GO:0007165 signal transduction
GO:0051301 cell division
GO:0042597 periplasmic space
GO:0016021 integral component of membrane
Proportion Compared to whole Genome
Avi/Caz - Higher Fitness
GO:0006094 gluconeogenesis
GO:0003723 RNA binding
GO:0016787 hydrolase activity
Avi/Caz - Lower Fitness
GO:0004888 transmembrane signaling receptor activity
GO:0016021 integral component of membrane
GO:0006935 chemotaxis
GO:0030288 outer membrane-bounded periplasmic space
GO:0005886 plasma membrane
GO:0016020 membrane
GO:0051301 cell division
GO:0000166 nucleotide binding
Proportion Compared to whole Genome
Tazobactam - Higher Fitness
GO:0008199 ferric iron binding
GO:0003755 peptidyl-prolyl cis-trans isomerase activity
GO:0030170 pyridoxal phosphate binding
Tazobactam - Lower Fitness
GO:0000270 peptidoglycan metabolic process
GO:0003774 cytoskeletal motor activity
GO:0051920 peroxiredoxin activity
GO:0004888 transmembrane signaling receptor activity
GO:0004497 monooxygenase activity
GO:0016021 integral component of membrane
Proportion Compared to whole Genome
Cycloserine - Higher Fitness
GO:0019700 organic phosphonate catabolic process
GO:0016779 nucleotidyltransferase activity
GO:0004725 protein tyrosine phosphatase activity
GO:0016740 transferase activity
Cycloserine - Lower Fitness
GO:0005507 copper ion binding
GO:0006807 nitrogen compound metabolic process
GO:0016620 oxidoreductase activity, NAD or NADP as acceptor
GO:0016853 isomerase activity
GO:0008360 regulation of cell shape
Proportion Compared to whole Genome
Fosfomycin - Higher Fitness
GO:0019700 organic phosphonate catabolic process
GO:0000155 phosphorelay sensor kinase activity
GO:0016491 oxidoreductase activity
Fosfomycin - Lower Fitness
GO:0001514 selenocysteine incorporation
GO:0000270 peptidoglycan metabolic process
GO:0016788 hydrolase activity, acting on ester bonds
GO:0016740 transferase activity
Proportion Compared to whole Genome
FR-900098 - Higher Fitness
GO:0008745 N-acetylmuramoyl-L-alanine amidase activity
GO:0008677 2-dehydropantoate 2-reductase activity
GO:0009116 nucleoside metabolic process
GO:0008168 methyltransferase activity
GO:0008080 N-acetyltransferase activity
GO:0016740 transferase activity
FR-900098 - Lower Fitness
GO:0005315 inorganic phosphate transmembrane transporter activity
GO:0004177 aminopeptidase activity
GO:0004601 peroxidase activity
GO:0005507 copper ion binding
GO:0004888 transmembrane signaling receptor activity
GO:0016614 oxidoreductase activity, acting on CH-OH group of donors
GO:0016853 isomerase activity
GO:0051920 peroxiredoxin activity
Proportion Compared to whole Genome
Bacitracin - Higher Fitness
GO:0015137 citrate transmembrane transporter activity
GO:0030638 polyketide metabolic process
GO:0000271 polysaccharide biosynthetic process
GO:0008137 NADH dehydrogenase (ubiquinone) activity
GO:0016740 transferase activity
Bacitracin - Lower Fitness
GO:0008839 4-hydroxy-tetrahydrodipicolinate reductase
GO:0004177 aminopeptidase activity
GO:0006979 response to oxidative stress
GO:0016853 isomerase activity
GO:0006355 regulation of transcription, DNA-templated
GO:0046872 metal ion binding
Proportion Compared to whole Genome

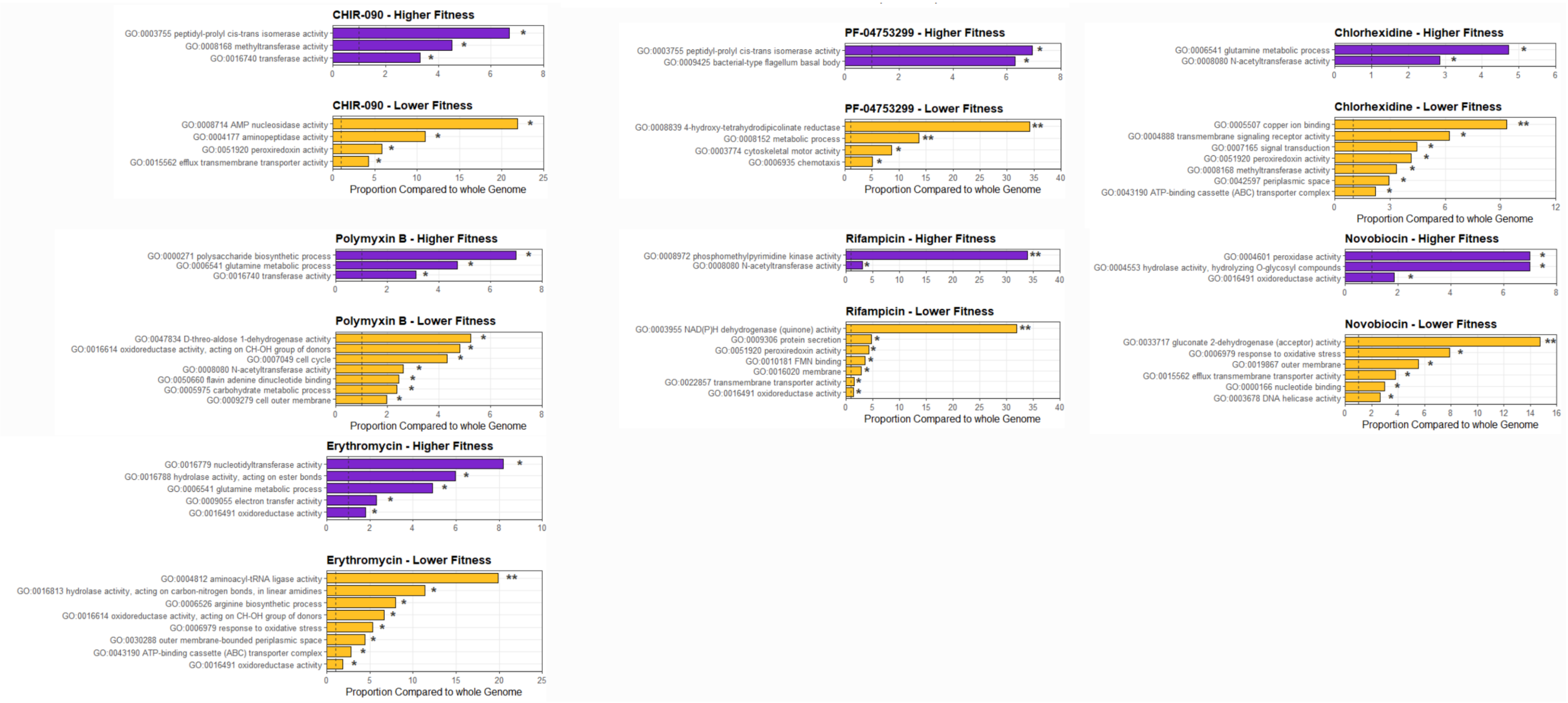

Supplementary Figure 7. GO term enrichment of genes affecting fitness in the presence of the antibiotic panel. Genes with fitness scores either above 0.5 or below -0.5 were used for GO term enrichment relative to the whole K56-2 genome (two-sided hypergeometric test without multiple testing correction) with GeneMerge 1.5 (Castillo-Davis et al. 2003). Enrichments from genes with positive and negative fitness scores are given in purple and gold, respectively. The dashed line indicates equal proportions in the genome and antibiotic condition. * $P < 0.05$, ** $P < 0.01$, *** $P < 0.001$.

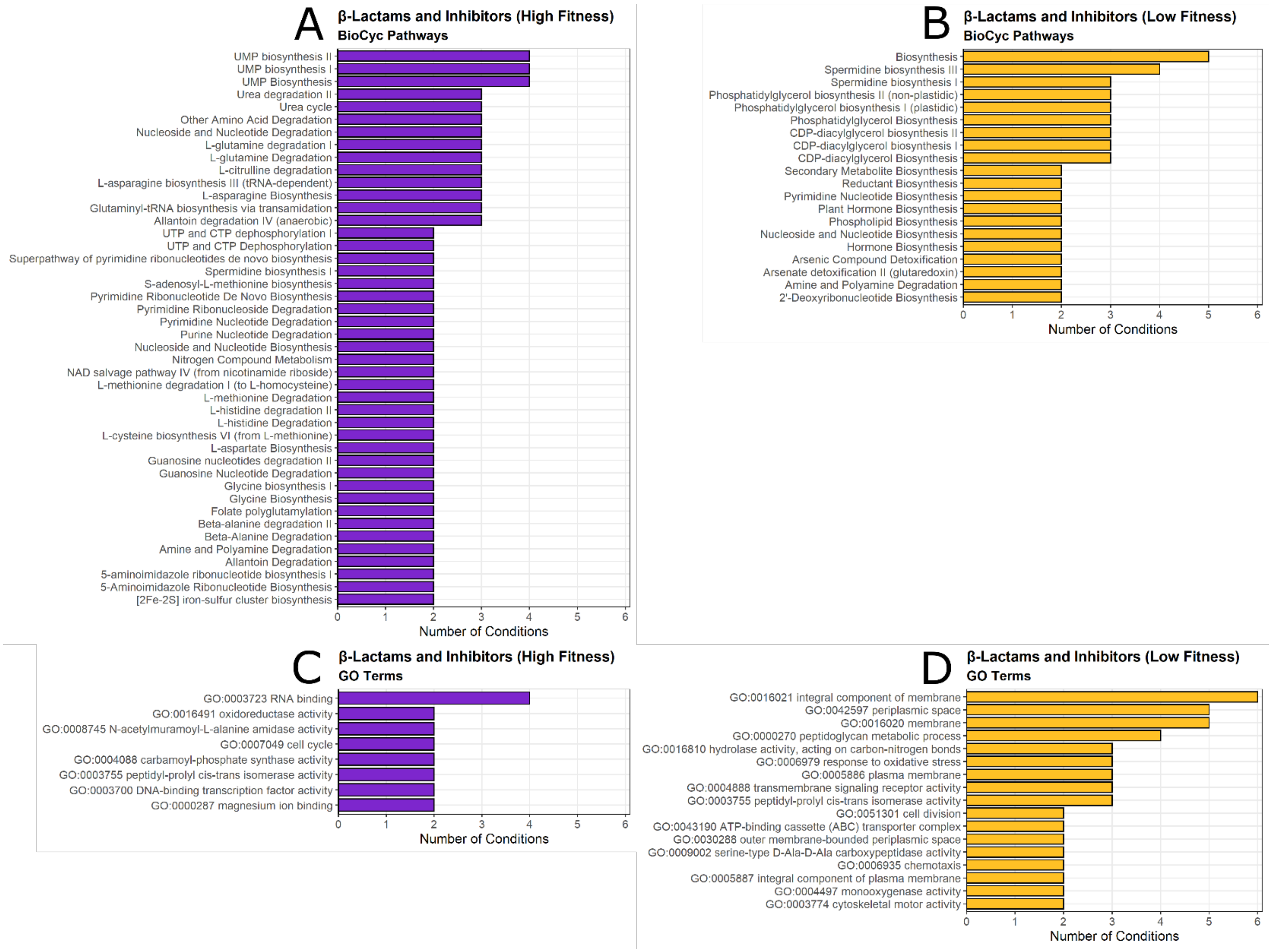

Supplementary Figure 8. Summary of BioCyc pathway and GO term enrichments from gene scores among the β-lactam and β-lactamase inhibitor conditions. Panels separate genes associated with higher fitness in the antibiotic condition (A and C) vs lower fitness (B and D), in addition to analysis by BioCyc pathways (A and B) or GO terms (C and D). Significant enrichments ($P < 0.05$)

from BioCyc pathways and GO terms were pooled from Supplementary Figures 6 (Fisher exact two-sided test without multiple testing correction) and 7 (two-sided hypergeometric test without multiple testing correction). Only those pathways and terms present in at least two antibiotic conditions are shown.

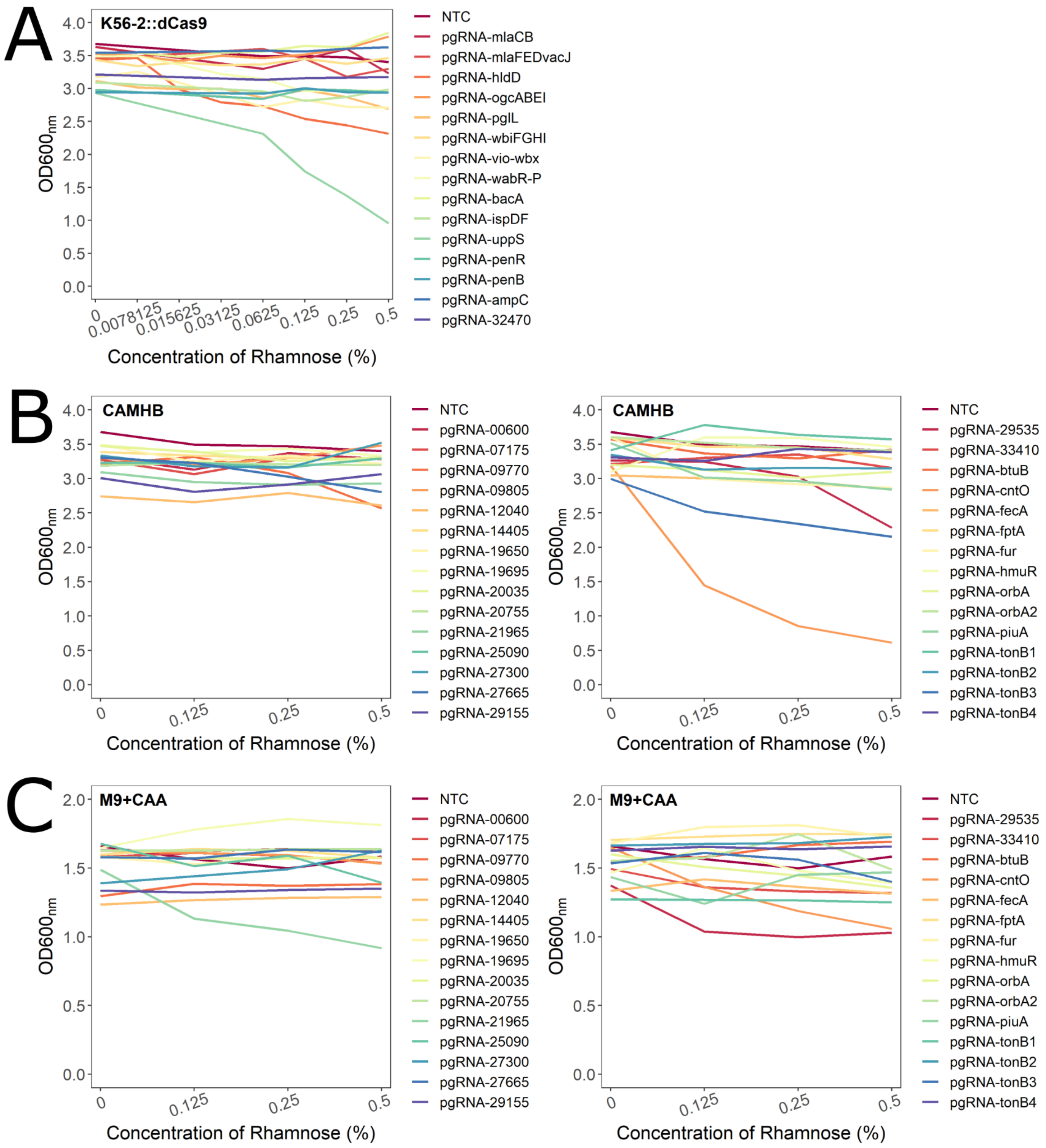
A
K56-2::dCas9
OD600nm
Concentration of Rhamnose (%)
0 0.0078125 0.015625 0.03125 0.0625 0.125 0.25 0.5
0.0 0.5 1.0 1.5 2.0 2.5 3.0 3.5 4.0
NTC
pgRNA-mlaCB
pgRNA-mlaFEDvacJ
pgRNA-hldD
pgRNA-ogcABEI
pgRNA-pglL
pgRNA-wbiFGHI
pgRNA-vio-wbx
pgRNA-wabR-P
pgRNA-bacA
pgRNA-ispDF
pgRNA-uppS
pgRNA-penR
pgRNA-penB
pgRNA-ampC
pgRNA-32470
B
CAMHB
OD600nm
Concentration of Rhamnose (%)
0 0.125 0.25 0.5
NTC
pgRNA-00600
pgRNA-07175
pgRNA-09770
pgRNA-09805
pgRNA-12040
pgRNA-14405
pgRNA-19650
pgRNA-19695
pgRNA-20035
pgRNA-20755
pgRNA-21965
pgRNA-25090
pgRNA-27300
pgRNA-27665
pgRNA-29155
CAMHB
NTC
pgRNA-29535
pgRNA-33410
pgRNA-btuB
pgRNA-cntO
pgRNA-fecA
pgRNA-fptA
pgRNA-fur
pgRNA-hmuR
pgRNA-orbA
pgRNA-orbA2
pgRNA-piuA
pgRNA-tonB1
pgRNA-tonB2
pgRNA-tonB3
pgRNA-tonB4
C
M9+CAA
0.0 0.5 1.0 1.5 2.0
M9+CAA

Supplementary Figure 9. Rhamnose dose-responses of select CRISPRi mutants constructed in this study. Cultures were grown with the indicated concentrations of rhamnose in A) LB, B) CAMHB, C) M9+CAA for 16 hours, the time at which the control strains reached the maximum OD_{600}, and then OD_{600} values were recorded. Each gene was targeted with two sgRNA, and one representative is shown here. If the targeted gene does not have a 4-letter name, the locus tag (preceded by K562_) is given. NTC = non-targeting control sgRNA. Values shown are averages of at least three biological replicates ($n \geq 3$); SD values are omitted for clarity.

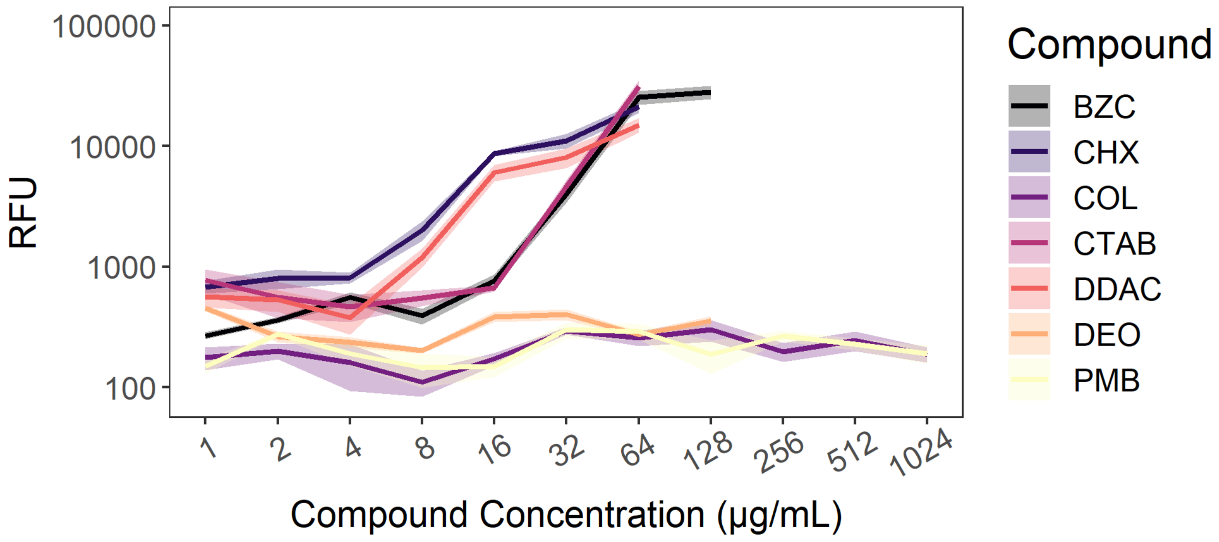

Supplementary Figure 10. Outer membrane permeability of K56-2 is increased by exposure to certain membrane disruptors. Exponential phase K56-2 was incubated with NPN and a concentration gradient of the indicated compounds. BZC = benzethonium chloride; CHX = chlorhexidine; COL = colistin; CTAB = cetrimonium bromide; DDAC = dodecyl dimethylammonium bromide; DEO = sodium deoxycholate; PMB = polymyxin B. Raw fluorescence values were blank corrected. The shading represents means ± SD of three biological replicates.

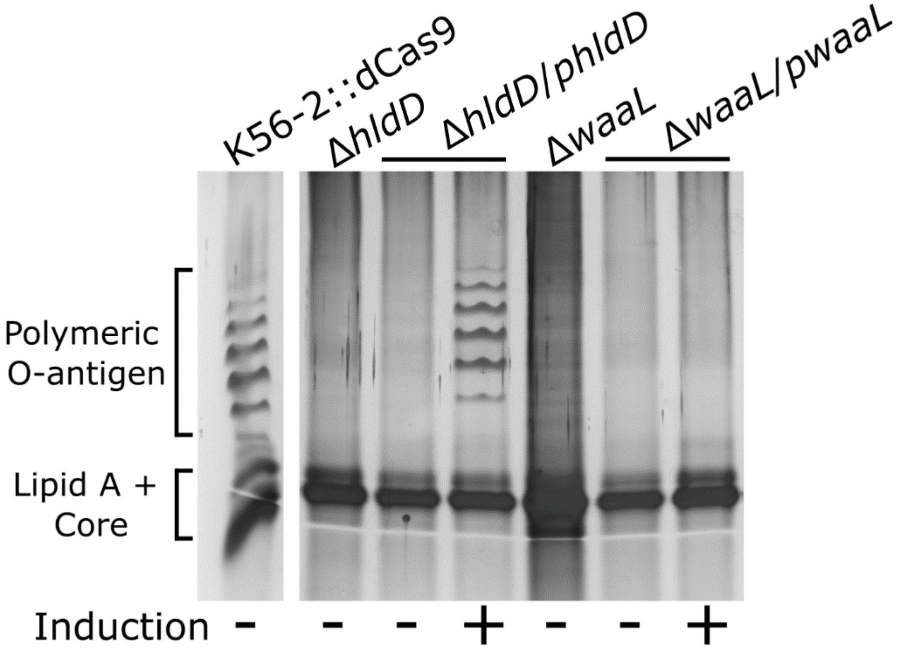

Supplementary Figure 11. Effect of deletion and complementation of *hldD* and *waaL* on O-antigen expression. These genes were deleted in the K56-2::dCas9 background. Silver-stained SDS polyacrylamide gels of LPS extracts from mid-exponential phase deletion and complementation mutants, grown for 4 hours with or without 0.05% rhamnose. Shown are different lanes from the same gel and representative blots from two biological replicates.

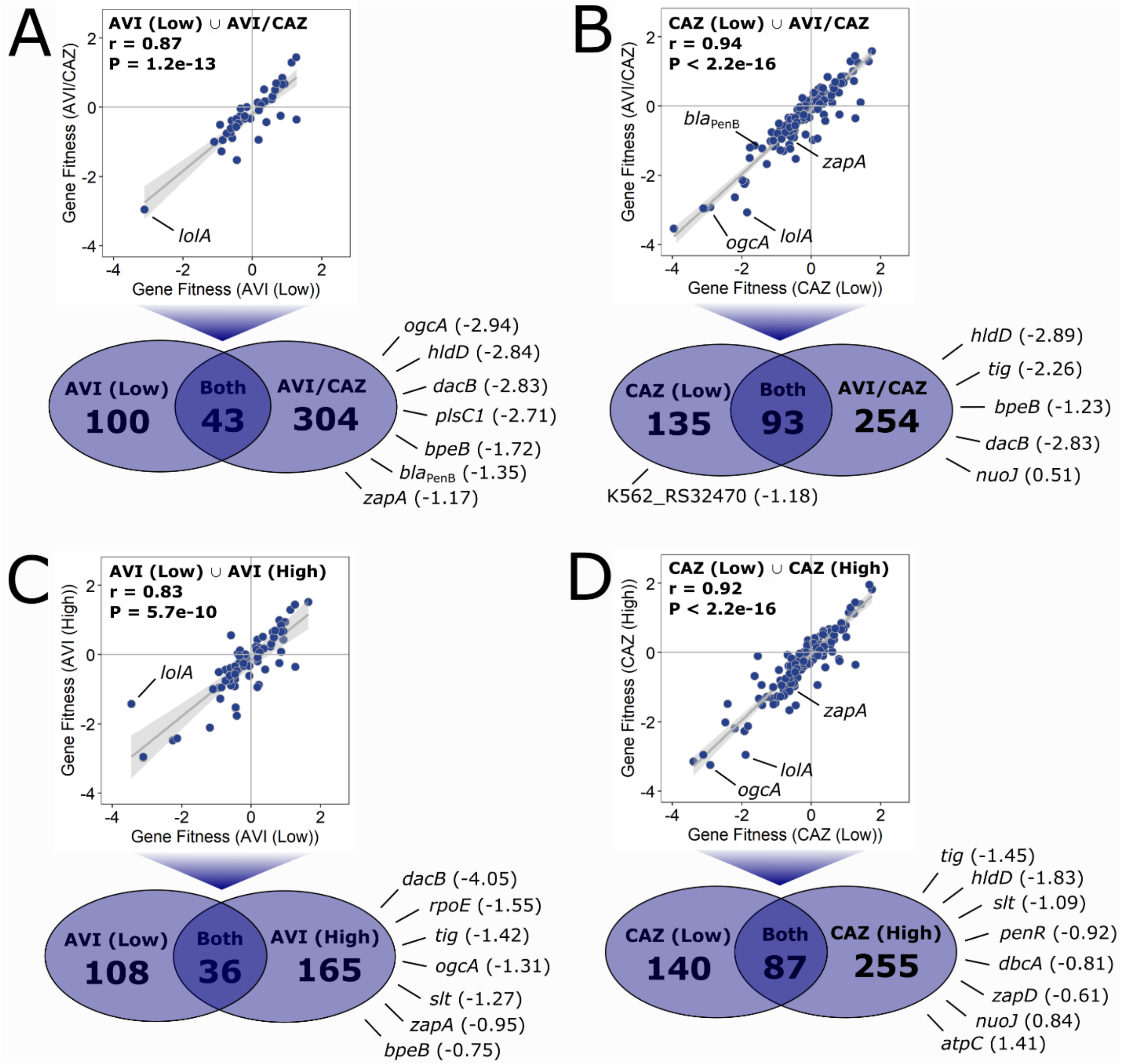

Supplementary Figure 12. Concentration- and combination-dependent chemical-genetic interactions with AVI/CAZ. Comparison of gene fitness scores between A) AVI/CAZ to AVI-L, B) AVI/CAZ to CAZ-L, C) AVI-H to AVI-L, and D) CAZ-H to CAZ-L. All genes with significantly different fitness scores greater than 0.5 or less than -0.5 relative to the DMSO control ($P < 0.05$, two-sided t-test) were used for comparison. For the genes that were common to both conditions, the scatter plots show a Pearson's correlation test (with 95% confidence interval in light grey; two-sided) and associated r and P-values. Each point shows the average gene fitness in a given condition from three biological replicates. In the Venn diagrams, fitness scores next to genes are relative to the DMSO control.

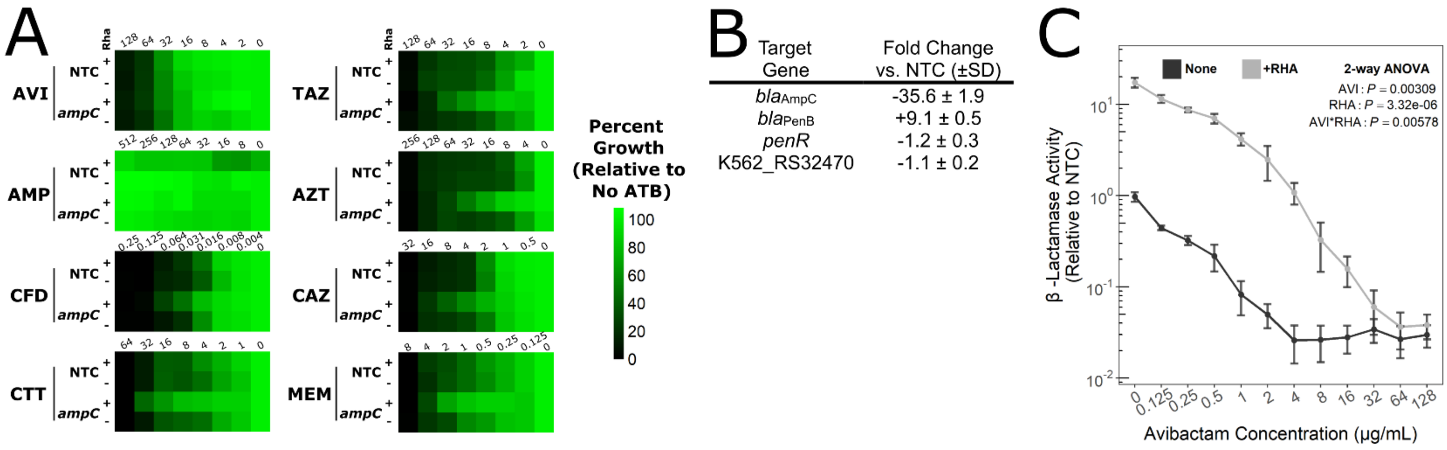

Supplementary Figure 13. Knockdown of *bla*AmpC reduces β-lactam susceptibility, but is still rescued by AVI. A) Antibiotic dose responses (µg/mL) of growth of the indicated mutants with or without CRISPRi induction with 0.5% rhamnose. Values are normalized to the OD_{600} of growth without antibiotic and are means of three biological replicates. NTC = non-targeting sgRNA control. B) Fold change in expression of β-lactamase-associated genes in the *bla*AmpC CRISPRi mutant induced with 0.5% rhamnose compared to the NTC mutant. Values are means ± SD of three biological replicates. C) Nitrocefin hydrolysis assay of the knockdown mutant in *bla*AmpC incubated with increasing concentrations of AVI with or without CRISPRi induction. Data was normalized to the NTC mutant. Values presented are means of four biological replicates, ± SD. Significance was determined by a two-way ANOVA.

Supplementary References

1. Darling, P., Chan, M., Cox, A. D. & Sokol, P. A. Siderophore production by cystic fibrosis isolates of *Burkholderia cepacia*. *Infection and immunity* **66**, 874–877 (1998).
2. Hogan, A. M., Rahman, A. S. M. Z., Lightly, T. J. & Cardona, S. T. A broad-host-range CRISPRi toolkit for silencing gene expression in *Burkholderia*. *ACS Synth. Biol.* **8**, 2372–2384 (2019).
3. Bloodworth, R. A. *et al.* Draft genome sequences of *Burkholderia contaminans*, a *Burkholderia cepacia* complex species that is increasingly recovered from cystic fibrosis patients. *Genome announcements* **3**, 10.1128/genomeA.00766-15 (2015).
4. Mahenthiralingam, E. *et al.* Diagnostically and experimentally useful panel of strains from the *Burkholderia cepacia* complex. *Journal of clinical microbiology* **38**, 910–913 (2000).
5. Meselson, M. & Yuan, R. DNA restriction enzyme from E. coli. *Nature* **217**, 1110–1114 (1968).
6. Miller, V. L. & Mekalanos, J. J. A novel suicide vector and its use in construction of insertion mutations: osmoregulation of outer membrane proteins and virulence determinants in *Vibrio cholerae* requires *toxR*. *Journal of Bacteriology* **170**, 2575–2583 (1988).
7. Aubert, D. F., Hamad, M. A. & Valvano, M. A. A markerless deletion method for genetic manipulation of *Burkholderia cenocepacia* and other multidrug-resistant gram-negative bacteria. *Methods Mol Biol* **1197**, 311–327 (2014).
8. Hamad, M. A., Skeldon, A. M. & Valvano, M. A. Construction of aminoglycoside-sensitive *Burkholderia cenocepacia* strains for use in studies of intracellular bacteria with the gentamicin protection assay. *Applied and Environmental Microbiology* **76**, 3170–3176 (2010).

9. Flannagan, R. S., Linn, T. & Valvano, M. A. A system for the construction of targeted unmarked gene deletions in the genus *Burkholderia*. *Environmental microbiology* **10**, 1652–1660 (2008).
10. Cardona, S. T., Mueller, C. & Valvano, M. A. Identification of essential operons in *Burkholderia cenocepacia* with a rhamnose inducible promoter. *Applied and Environmental Microbiology.* **72**, 2547–2555 (2006).
11. Bloodworth, R. A. M., Gislason, A. S. & Cardona, S. T. *Burkholderia cenocepacia* conditional growth mutant library created by random promoter replacement of essential genes. *MicrobiologyOpen* **2**, 243–258 (2013).
12. Figurski, D. H. & Helinski, D. R. Replication of an origin-containing derivative of plasmid RK2 dependent on a plasmid function provided in trans. *Proceedings of the National Academy of Sciences of the United States of America* **76**, 1648–1652 (1979).
13. Hogan, A. M., Jeffers, K. R., Palacios, A. & Cardona, S. T. Improved dynamic range of a rhamnose-inducible promoter for gene expression in *Burkholderia* spp. *Appl Environ Microbiol* **87**, e0064721 (2021).
14. Luo, H. & Gao, F. DoriC 10.0: an updated database of replication origins in prokaryotic genomes including chromosomes and plasmids. *Nucleic Acids Res* **47**, D74–D77 (2019).
