# Supplementary material for: Profiling cell envelope-antibiotic interactions reveals vulnerabilities to β-lactams in a multidrug-resistant bacterium
Source: Nat Commun. 2023 Aug 9;14:4815. doi: 10.1038/s41467-023-40494-5 (PMC10412643; doi:10.1038/s41467-023-40494-5)
Supplement: Supplementary file 5 — Reporting Summary [file 41467_2023_40494_MOESM5_ESM.pdf]

nature portfolio

Corresponding author(s): Silvia T. Cardona

Last updated by author(s): 2023/07/15

Reporting Summary

Nature Portfolio wishes to improve the reproducibility of the work that we publish. This form provides structure for consistency and transparency in reporting. For further information on Nature Portfolio policies, see our Editorial Policies and the Editorial Policy Checklist.

Statistics

For all statistical analyses, confirm that the following items are present in the figure legend, table legend, main text, or Methods section.

n/a	Confirmed	
☐	☒	The exact sample size (n) for each experimental group/condition, given as a discrete number and unit of measurement
☐	☒	A statement on whether measurements were taken from distinct samples or whether the same sample was measured repeatedly
☐	☒	The statistical test(s) used AND whether they are one- or two-sided<br>*Only common tests should be described solely by name; describe more complex techniques in the Methods section.*
☒	☐	A description of all covariates tested
☐	☒	A description of any assumptions or corrections, such as tests of normality and adjustment for multiple comparisons
☐	☒	A full description of the statistical parameters including central tendency (e.g. means) or other basic estimates (e.g. regression coefficient) AND variation (e.g. standard deviation) or associated estimates of uncertainty (e.g. confidence intervals)
☐	☒	For null hypothesis testing, the test statistic (e.g. F, t, r) with confidence intervals, effect sizes, degrees of freedom and P value noted<br>*Give P values as exact values whenever suitable.*
☒	☐	For Bayesian analysis, information on the choice of priors and Markov chain Monte Carlo settings
☒	☐	For hierarchical and complex designs, identification of the appropriate level for tests and full reporting of outcomes
☐	☒	Estimates of effect sizes (e.g. Cohen's d, Pearson's r), indicating how they were calculated

Our web collection on statistics for biologists contains articles on many of the points above.

Software and code

Policy information about availability of computer code

Data collection	Illumina platforms were managed by the Illumina Experiment Manager v1.18; Biotek plate readers were controlled with Gen5 version 3.10; micrographs were acquired with Zen 3.2; Bio-rad Image Lab Touch version 2.4.0.03 was used for LPS gel imaging; StepOnePlus version 2.3 (Applied Biosystems) was used to operate the qPCR thermocycler.
Data analysis	FASTX toolkit v0.0.14 (http://hannonlab.cshl.edu/fastx_toolkit/), simple shell commands with Unix and Python 3.7, and scripts from https://bitbucket.org/berkeleylab/feba/src/master/ and https://github.com/DuttonLab/RB-TnSeq-Microbial-interactions were used to process sequencing read data. Enrichment on gene sets was performed with BioCyc smart tables built-in statistics tools and GeneMerge 1.5 (for GO categories). Pearson's correlation was performed with base R stats test cor(). RStudio v2022.07.2+576 was used as the environment for R. SynergyFinder (https://synergyfinder.fimm.fi/) was used to process antibiotic interactions. StepOnePlus version 2.3 (Applied Biosystems) software was used to calculate qRT-PCR primer efficiency. ChemMine Tools was used to obtain molecular properties from the antibiotic panel. The prcomp function in R was used for PCA on the antibiotic properties.

For manuscripts utilizing custom algorithms or software that are central to the research but not yet described in published literature, software must be made available to editors and reviewers. We strongly encourage code deposition in a community repository (e.g. GitHub). See the Nature Portfolio guidelines for submitting code & software for further information.

Data

Policy information about availability of data

All manuscripts must include a data availability statement. This statement should provide the following information, where applicable:

- Accession codes, unique identifiers, or web links for publicly available datasets
- A description of any restrictions on data availability
- For clinical datasets or third party data, please ensure that the statement adheres to our policy

Raw sequencing data is available from the NCBI Sequencing Read Archive (SRA) under the BioProject ID PRJNA859150. All gene fitness scores are available in a spreadsheet in Supplementary Data 1. Source data to reproduce figures are also given with this manuscript. Publicly available databases and servers were used for gene annotations: BioCyc (https://biocyc.org/), EggNOG-mapper v2 (http://eggnog-mapper.embl.de/), GO (http://geneontology.org/), and UniProt (https://www.uniprot.org/). The closed K56-2 genome sequence was used with accession GCF_014357995.1.
The Shiny app graphical user interface is freely available at https://cardonalab.shinyapps.io/bcc_interaction_viewer/ , and the source code is available at https://github.com/cardonalab/Shiny-Bcc-ATB-Viewer

Research involving human participants, their data, or biological material

Policy information about studies with human participants or human data. See also policy information about sex, gender (identity/presentation), and sexual orientation and race, ethnicity and racism.

Reporting on sex and gender	Research does not involve human subjects
Reporting on race, ethnicity, or other socially relevant groupings	Research does not involve human subjects
Population characteristics	Research does not involve human subjects
Recruitment	Research does not involve human subjects
Ethics oversight	Research does not involve human subjects

Note that full information on the approval of the study protocol must also be provided in the manuscript.

Field-specific reporting

Please select the one below that is the best fit for your research. If you are not sure, read the appropriate sections before making your selection.

[x] Life sciences   [ ] Behavioural & social sciences   [ ] Ecological, evolutionary & environmental sciences

For a reference copy of the document with all sections, see nature.com/documents/nr-reporting-summary-flat.pdf

Life sciences study design

All studies must disclose on these points even when the disclosure is negative.

Sample size	Sample sizes were selected to allow appropriate statistical testing within the limits of funding and practical feasibility. All experiments were performed with at least three biological replicates.
Data exclusions	No datapoints were excluded from analysis.
Replication	Experiments with the transposon mutant library were performed with an inoculum large enough to allow for at least 75 CFU/mutant per tube, thus reducing random effects. The scripts used to process the BarSeq data aggregated effects across mutants in each gene, reducing the variability and increasing the statistical power. After processing the high-throughput mutant exposure experiments, genes of interest were validated in follow-up experiments with defined mutants.<br>Experimental findings with deletion/CRISPRi mutants from the first replicate were validated by 2 other replicates on different days. If the variation was larger, then additional replicates were performed.
Randomization	Allocation of cells and cultures into experimental groups was random. Transposon mutants were also collected randomly from the agar plates and pooled. Edge-effects in 96-well plates were minimized by sealing the plate edges with parafilm and incubation in a humidity controlled incubator.
Blinding	Investigators were not blinded and blinding was not relevant to this study design; the data collected was objective and quantitative. However, samples that were sent away for processing (Illumina sequencing pools, ICP-MS, and plasmid sequencing) were blinded by giving each sample alphanumeric codes. While investigators processing the data were not blinded to sample names, other investigators that had access to the data evaluated the results independently and obtained the same conclusions.

Reporting for specific materials, systems and methods

We require information from authors about some types of materials, experimental systems and methods used in many studies. Here, indicate whether each material, system or method listed is relevant to your study. If you are not sure if a list item applies to your research, read the appropriate section before selecting a response.

Materials & experimental systems

n/a	Involved in the study	
☒	☐	Antibodies
☒	☐	Eukaryotic cell lines
☒	☐	Palaeontology and archaeology
☒	☐	Animals and other organisms
☒	☐	Clinical data
☒	☐	Dual use research of concern
☒	☐	Plants

Methods

n/a	Involved in the study	
☒	☐	ChIP-seq
☒	☐	Flow cytometry
☒	☐	MRI-based neuroimaging
